# Inhibition of HOX/PBX Dimers as a Potential Therapeutic Strategy in Breast Cancer Subtypes Including Triple Negative Breast Cancer

**DOI:** 10.3390/cimb48080800

**Published:** 2026-08-07

**Authors:** Richard Morgan, Guy Simpson, Einthavy Arunachalam, Hardev Pandha

**Affiliations:** 1 School of Medicine and Biosciences, University of West London, London W5 5RF, UK; 2 Faculty of Health and Medical Sciences, University of Surrey, Guildford GU2 7XH, UK; g.simpson@surrey.ac.uk (G.S.); h.pandha@surrey.ac.uk (H.P.); 3 Engelward Laboratory, Department of Biological Engineering, Massachusetts Institute of Technology, Cambridge, MA 02139, USA; einthavy@mit.edu

**Keywords:** triple negative breast cancer, HOX, PBX, transcription factor, apoptosis

## Abstract

Triple negative breast cancer (TNBC) continues to have a poor prognosis relative to other forms of this disease. Previous studies have shown that the HOX family of transcription factors generally show increased expression in breast cancer and may have a primarily pro-oncogenic role. In this study, we assessed the sensitivity of a range of TNBC-derived cell lines to an inhibitor of HOX protein function, HTL-001, which blocks the interaction between HOX proteins and the Pre-B-cell Leukaemia Homeobox (PBX) cofactor. The sensitivity of cell lines was measured by MTS viability assays, and gene expression by RT-qPCR. Combination studies were performed with epigenetic modifiers (5-azacytidine (5-aza), Trichostatin A (TSA)) and standard-of-care chemotherapeutic drugs including Paclitaxel. A mouse tumour flank model of MDA-MB-231 cells was used to assess response to HTL-001, paclitaxel, or combination therapy. All the cell lines exhibited high levels of HOX dysregulation compared to an immortalised line derived from normal breast cells, and greater sensitivity to HTL-001-induced apoptosis. Epigenetic changes have previously been shown to be key modulators of HOX expression and, correspondingly, we show that reversing epigenetic changes in these cell lines significantly alters HOX expression and generally reduces sensitivity to HTL-001. In addition, HTL-001 shows synergistic interactions with several established chemotherapeutic agents in vitro. We further demonstrate that HTL-001 can significantly reduce tumour growth in a mouse model of TNBC. Our findings indicate that HOX/PBX dimers are a potential therapeutic target in this cancer.

## 1. Introduction

Despite enormous advances greatly improving the prognosis and quality of life for women suffering from breast cancer, current treatments face challenges including developing drug resistance (limited duration of response), the heterogeneity of tumours, managing severe side effects, selecting optimal treatments from a growing number of therapies, and the need for more accessible and personalised approaches. In particular, specific subtypes such as triple negative breast cancer (TNBC) are more aggressive and have fewer treatment options, highlighting the need for alternative treatments.

It is now well established that *HOX* genes, which encode a family of homeodomain-containing transcription factors within four discrete clusters (A–D) on different chromosomes, are highly dysregulated in cancer. Both tumour-suppressor and pro-oncogenic roles have been described for *HOX* genes, although generally only the latter are supported by direct experimental approaches, rather than associative studies [1]. This is particularly well established in breast cancer, in which several *HOX* genes have been shown to have specific roles in cancer progression. For example, *HOXB5* and *HOXB7* promote Fibroblast Growth Factor 2 (FGF2) expression [2] and increase epithelial to mesenchymal transition (EMT) [3], and *HOXB7* increases resistance to Tamoxifen through the upregulation of the Epidermal Growth Factor Receptor (EGFR) [4], as does *HOXA5* through an increase in PI3K/AKT signalling [5]. The function of HOX transcription factors is modified by and sometimes depends on binding to other transcription factors, including the Pre-B-cell Leukaemia Homeobox (PBX) and Myeloid Ecotropic Viral Integration Site 1 Homologue (MEIS) proteins that are members of the Three Amino Acid Loop Extension (TALE) family [6], which are also frequently dysregulated in cancer [7].

The pro-oncogenic function of HOX proteins make them a potential therapeutic target in cancer. However, this has generally proved difficult to achieve due to the high level of functional redundancy within the 39 members of this family [8,9], and more broadly because of the complexities of targeting protein/protein interactions [10]. An alternative approach is to target the interaction between HOX proteins and the PBX transcription factors as HOX/PBX heterodimers have more specific DNA binding sequences and a greater affinity for these sites [11]. Competitive inhibition of HOX/PBX binding has been achieved using a cell-penetrating peptide, HXR9, which mimics the hexapeptide sequence in HOX proteins that binds into a pocket in PBX [12]. HXR9 has been shown to cause apoptosis in a range of cancers [1], including breast cancer [12]. The second peptide inhibitor of HOX/PBX binding, HTL-001, has more recently been described, and is also a mimic of the hexapeptide region but is more effective at inducing apoptosis [13].

Although the efficacies of HXR9 and most recently HTL-001 have been widely studied, to date there have been no comprehensive studies of potential synergies between HOX/PBX inhibition and established cancer therapeutics. In this study, we have used TNBC-derived cell lines to assess the possible drug synergies between HTL-001 and Paclitaxel, 5-Fluorouracil (5-FU) and Palbociclib, which are used to treat TNBC and metastatic breast cancer in the clinic [14,15], and metformin, which is the initial drug choice for type 2 diabetic patients, but could potentially be repurposed for TNBC patients [16]. In addition, we examined the effects of agents that reverse epigenetic changes in the form of methylation and histone modification on HTL-001 sensitivity and HOX gene expression. This reveals extensive synergistic interactions between HTL-001 and these therapeutics with an optimal concentration of HTL-001 below its IC50 as a single agent, and lower doses of the conventional drugs, potentially lowering toxicity.

## 2. Materials and Methods

### 2.1. Cell Lines

The cell lines MCF-7 (accession number RRID: CVCL_0031), ZR-75-1 (accession number RRID: CVCL_0588), MDA-MB-231 (accession number RRID: CVCL_0062), BT-20 (accession number RRID: CVCL_0178), and SK-BR-3 (accession number RRID: CVCL_0033) were purchased from the American Type Culture Collection (ATCC, Manassas, VA, USA), and MDA-MB-231-BR (accession number RRID: CVCL_A0YY) was kindly gifted by Dr Patricia S. Steeg (National Cancer Institute, Bethesda, MD, USA). All cell lines commercially available from ATCC were authenticated and cell-validated using short tandem repeat (STR) profiling and comparatively analysed to authenticated STR profiles of LGC standards (UK); a threshold of ≥80% match between the STR profiles of the cultured cell lines and of ATCC-LGC standards were considered as validated. MycoAlertTM PLUS Mycoplasma Detection Kit (Lonza, Walkersville, MA, USA, LT07-710) was used monthly to ensure all cell lines were mycoplasm-free before any experimental use of the cell lines, as well as before and after cryopreservation in liquid nitrogen.

All cell lines, except MCF10a, were cultured in ATCC-recommended or supplier-recommended media, supplemented with 100 U/mL Penicillin (Sigma-Aldrich, Gillingham, UK, P0781), 10 μg/mL Streptomycin (Sigma-Aldrich, P0781), 2 mM L-glutamine (Sigma-Aldrich, G7513) and 10% Foetal Bovine Serum (FBS, Gibco, Thermo Fisher Scientific, Altrincham, UK, 10270). MCF10a media was supplemented with 5% Horse Serum (Thermo Fisher Scientific, 16050122), 20 ng/mL Epidermal Growth Factor (EGF, Peprotech, London, UK, AF-100-15), 0.5 mg/mL Hydrocortisone (Sigma-Aldrich, H0888), 100 ng/mL Cholera Toxin (Sigma-Aldrich, C8052), 10 μg/mL Insulin (Sigma-Aldrich, I1882), 100 U/mL Penicillin (Sigma-Aldrich, P0781) and 10 μg/mL Streptomycin (Sigma-Aldrich, P0781).

### 2.2. Peptide Synthesis

Peptides were synthesised by Sigma-Aldrich using conventional column-based chemistry to ≥95% purity as a powder formulation, and solubilised in PBS to a stock concentration of 50 mM and stored at −20 °C.

### 2.3. Cell Survival Analysis by MTS Assay

The viability of cancer cells in the absence and presence of various chemotherapeutic agents and targeted agents was evaluated using the CellTiter 96^®^ AQueous One Solution Cell Proliferation Assay (MTS, Promega, Southampton, UK, G3580) in accordance with the manufacturer’s instructions. The cell survival (%) values of all technical and biological replicates were interpolated against each drug concentration into GraphPad Prism 7 Software (GraphPad Prism, San Diego, CA, USA) and non-linear regression (curve fit) analysis was used to calculate the IC50 and IC25 specific to each chemotherapeutic agent and targeted therapy. The IC25 and IC50 define the inhibitor concentrations that reduced the cell survival to 25% and 50% of the maximal control (untreated cells) value, respectively.

### 2.4. RNA Extraction and Quantification

For baseline gene expression analysis of cell lines, 1 × 10^6^ cells were counted and separated from cell suspensions into RNase-free Eppendorf tubes. For gene expression analysis of cell lines under specific conditions such as various drug treatments, reversal of epigenetic modifications or transient RNA silencing, cells were counted and plated in 6-well plates at seeding densities of 4 × 10^5^ cells per well. Three wells were used per condition and each experimental design was repeated 3 times biologically. Cells were extracted from wells using StemPro™ Accutase™ Cell Dissociation Reagent (Thermo Fisher Scientific) and collected in RNase-free Eppendorf tubes. RNA was extracted using the RNeasy Plus Mini Kit (Qiagen, Manchester, UK, 74134) following the manufacturer’s instructions. The resulting RNA in aqueous solution was quantified and assessed for purity using a NanoDrop^®^ Spectrophotometer ND-1000 (Thermo Fisher Scientific).

### 2.5. cDNA Synthesis and Semi-Quantitative PCR

cDNA was synthesised by reverse transcription of 1000 ng RNA using the SuperScript™ First-Strand Synthesis System for RT-PCR kit (Invitrogen, Carlsbad, CA, USA, 11904018), according to the manufacturer’s instructions. PCR was performed using the Stratagene Mx3000P qPCR System (Agilent Technologies, Santa Clara, CA, USA) with SYBR^®^ Green JumpStart™ Taq ReadyMix™ (Sigma-Aldrich, S4438). *Βeta-actin* or *Glyceraldehyde-3-Phosphate Dehydrogenase* (*GAPDH*) were used as internal controls to which the expression of other genes was normalised. The cycling conditions, primer sequences and data analysis were as previously described [14].

### 2.6. Treatments to Reverse Epigenetic Modifications

Inhibitors of commonly observed epigenetic modifications were used to reverse epigenetic gene silencing or epigenetic overexpression. 5-azacytidine (5-aza), alone and in combination with Trichostatin A (TSA), was used to reverse DNA methylation and histone deacetylation, respectively. Before treatment, untreated cells were replenished with fresh complete media whilst all treatment wells were treated with 10 μM 5-aza (Sigma-Aldrich, A2385) at 37 °C for 48 h. After 48 h of incubation with 5-aza, cells were further incubated with 5-aza for 24 h, whilst wells to be treated concurrently with these reagents were treated with 10 μM 5-aza and 0.5 μM TSA (Selleckchem, London, UK, S1045) at 37 °C for 24 h.

### 2.7. Annexin V/7-AAD Staining for Cell Viability

Annexin V/7-AAD staining was performed to assess cell viability and apoptotic cell death in cultured cell lines. Cells were seeded at 3 × 10^5^ cells per well in 6-well plates and allowed to attach for 24 h at 37 °C before treatment with vehicle control or HTL-001 for 2 h. Following treatment, cells were harvested using trypsin, collected by centrifugation, and stained with the Annexin V–PE apoptosis detection kit (BD Biosciences, Heidelberg, Germany) according to the manufacturer’s instructions. Stained cells were analysed on a MACSQuant flow cytometer (Miltenyi Biotec, Woking, UK), and data were processed using MACSQuantify version 3.1 (Miltenyi Biotec) software to quantify viable, early apoptotic, late apoptotic, and necrotic populations.

### 2.8. Co-Localisation Immunofluorescence

Co-localisation immunofluorescence was performed in cells seeded at 1 × 10^4^ cells per well in 8-well chamber slides and cultured for 24 h at 37 °C before treatment with vehicle control or drug for 2 h. Cells were fixed in 3.7% paraformaldehyde for 15 min, permeabilised in 0.5% Triton X-100 for 5 min, and blocked with 5% bovine serum albumin (BSA). Slides were incubated overnight at 4 °C with primary antibodies diluted in blocking buffer, followed by incubation with appropriate fluorophore-conjugated secondary antibodies. The primary antibodies used were PBX1 Antibody (Cell Signalling Technology, Danvers, MA, USA, 4342, 1:1000), anti-HOXA1 (Santa Cruz Biotechnology, Santa Cruz, CA, USA, sc-17146, 1:1000), anti-HOXB7 (Santa Cruz Biotechnology, sc-81292, 1:1000) anti-HOXC4 (Santa Cruz Biotechnology, sc-398460, 1:1000) and anti-HOXC9 (Santa Cruz Biotechnology, sc-81100, 1:1000). Secondary antibodies were FITC-conjugated goat anti-mouse IgG (Invitrogen, F2761, 1:50) and TRITC-conjugated goat anti-rabbit IgG (Invitrogen, T2769, 1:50). After removal of the chambers, slides were mounted and counterstained with SlowFade Gold Antifade Mountant containing DAPI (Thermo Fisher Scientific, S36939). Fluorescence signals were visualised using a Nikon A1M laser-scanning confocal microscope (Nikon Instruments Inc., Melville, NY, USA) equipped with a DS-Qi1 camera, with FITC (green) and TRITC (red) emission channels used for co-localisation analysis.

### 2.9. Western Blot Analysis

Western blotting was performed to assess protein expression and phosphorylation in treated cell lines. Cells were seeded at 3 × 10^5^ cells per well in 6-well plates and cultured for 24 h at 37 °C before exposure to vehicle control or drug for 2 h. Following treatment, cells were harvested by trypsinisation, pelleted by centrifugation, and lysed in RIPA lysis buffer (Thermo Fisher Scientific, 89900) supplemented with HALT protease and phosphatase inhibitor cocktail (Thermo Fisher Scientific, 78440). Equal amounts of protein (20 µg per sample) were resolved on NuPAGE 4–12% Bis-Tris gels (Thermo Fisher Scientific, NP0322) and transferred to PVDF membranes using an iBlot transfer system (Thermo Fisher Scientific, IB31001). Membranes were blocked in 1% skimmed milk and incubated overnight at 4 °C with primary antibodies against c-Fos (Cell Signalling Technology, 2250, 1:1000), phospho-DUSP1/MKP1 (Cell Signalling Technology, 2857, 1:1000), EGR1 (Cell Signalling Technology, 4153, 1:1000), or GAPDH (Cell Signalling Technology, 5174, 1:2000). After washing, membranes were incubated for 1 h at room temperature with HRP-conjugated secondary antibodies (anti-rabbit IgG, Cell Signalling Technology, 7074, 1:2000; or anti-mouse IgG, Cell Signalling Technology, 7076, 1:2000). Protein bands were visualised using SuperSignal West Femto chemiluminescent substrate (Thermo Fisher Scientific, 34094) and imaged on a UVP BioDoc-It system.

### 2.10. In Vivo Subcutaneous Mouse Model

All in vivo studies were carried out under Project Licence number PBE74785E. The University of Surrey’s Animal Welfare and Ethical Review Body approved and oversaw all in vivo experiments.

**Figure 1:**
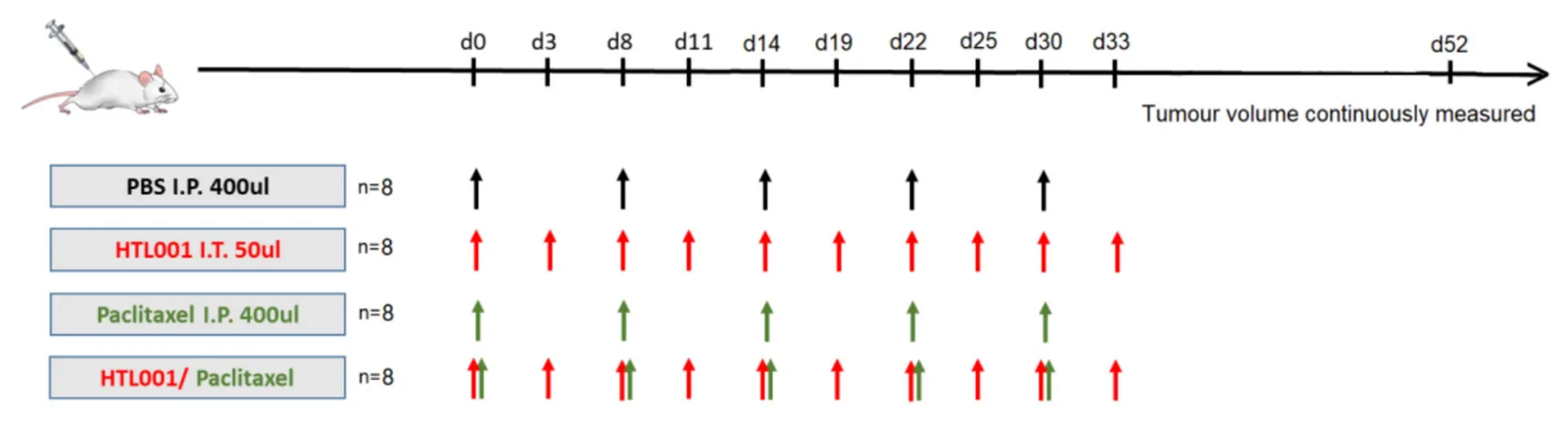
Experimental plan for in vivo model to assess tumour volume and overall survival.

In this study, 32 5-week old female BALBc nude mice (Charles River Laboratory, Margate, UK) were used. 100 μL of 5 × 10^6^ MDA-MB-231-BR cells suspended in 50% PBS/Matrigel solution (Sigma-Aldrich) were injected subcutaneously into the flank of each mouse. Tumours were left to establish for 2 weeks. Tumour volume was measured three times a week using vernier callipers and treatment was started once tumours had reached approximately 50–60 mm^3^. Mice were divided equally amongst 4 groups: 8 mice received PBS treatment only, 8 mice received HTL-001 at 20 mg/kg intratumorally, 8 mice received Paclitaxel at 10 mg/kg intraperitoneally and 8 mice received HTL-001 at 20 mg/kg intratumorally and Paclitaxel at 10 mg/kg intraperitoneally. HTL-001 was prepared fresh from powder in sterile PBS and Paclitaxel was prepared fresh from solution in sterile PBS. The following day a single 50 μL injection of HTL-001 or 400 μL injection of Paclitaxel was administered intratumorally or intraperitoneally, respectively, at the appropriate concentrations. PBS mice received 50 μL PBS injection only. HTL-001 was administered twice weekly and Paclitaxel was administered once a week. Treatment was administered for 5 consecutive weeks during which the tumour volume was measured every 2–3 days. Tumour volume graphs were created for each treatment group and plotted against each other for comparison. The dosing regime is summarised in Figure 1.

### 2.11. Statistical Analysis

GraphPad^®^ Prism 7 (GraphPad Software, Inc., Boston, MA, USA) was used to analyse and graphically present data. The IC50 of HTL-001 and each chemotherapeutic drug was calculated from plotting the dose response curve in GraphPad^®^ Prism 7 and determined by non-linear regression from an [inhibitor] vs. response–variable slope (four parameters). When there were two independent variables or two or more groups of data that were non-parametrically distributed, two-way analysis of variance (ANOVA) with Dunnett’s test or Kruskal–Wallis tests for multiple comparison were used to analyse the data for statistical significance. All numeric values were calculated as the mean of three independent biological experiments with error bars that represented the standard deviation (SD) of the mean. The *p* value was used to define significance; when the comparative analysis between untreated vs. treated groups or between different treatment groups gave *p* values < 0.05, the difference was deemed significantly different. Different levels of significance were depicted by symbol indices of * for a *p* value < 0.05, ** for a *p* value < 0.01, *** for a *p* value < 0.001 and **** for a *p* value of <0.0001. Statistical difference between 2 or more defined means, such as that of in vivo growth curve analysis, was calculated by multiple unpaired Student’s *t*-tests.

To assess the interaction of treating cancer cells with a combination of HTL-001 and a chemotherapeutic agent, at non-fixed dose ratios, the Bliss Independence Model was used. On the basis of both drugs working independently, the combined inhibitory effect is predictively calculated using the complete additivity probability theory, which states Eexp = (FDRUG A + FDRUG B) − (FDRUG A × FDRUG B). Bliss analysis examined additive, synergistic and antagonist effects of combined therapy and applied a second equation to assess this; ΔE = Eobs − Eexp. When the observed effect (Eobs) was equal to the expected effect (Eexp), the difference in effect, expressed as ΔE, was equal to zero and the combined therapy was of Bliss Independence; an additive effect. When ΔE was greater than zero, the combined therapy was deemed synergistic and more therapeutic. When ΔE was less than zero, the combined therapy was deemed antagonistic and of less therapeutic benefit. The interaction between each combination of drugs was expressed in a colour-coded contour map of the ΔE values.

## 3. Results

### 3.1. Evaluation of HOX, PBX and MEIS Gene Expression in Breast Cancer Cell Lines

*HOX* and *TALE* gene expression has previously been shown to be dysregulated in breast cancer cell lines, compared to that in normal breast cell lines [12]. In order to provide a more comprehensive understanding of this, we analysed the expression of all 39 *HOX* genes and the *PBX* and *MEIS* genes in a range of breast cancer cell lines, together with an immortalised non-tumourigenic breast cell line, MCF10a. Notably, this revealed extremely high levels of *HOXA* expression in MCF10a (Supplemental Figure S1), whereas *HOXA* expression is far lower in breast cancer cell lines, especially in those derived from more aggressive molecular subclassifications such as MDA-MB-231/MDA-MB-231-BR and SK-BR-3, which belong to TNBC and HER2-enriched subtypes, respectively (Supplemental Figure S1). For the *HOXB* and *HOXC* clusters, a dysregulation in gene expression can be seen with shifts in expression towards more anterior or central *HOX* genes in breast cancer cell lines. With *HOXB* genes, there is very slight increase in expression in central posterior genes, specifically *HOXB5* (*p* < 0.005 for MDA-MB-231, MDA-MB-231-BR and SK-BR-3 compared to MCF10a) (Supplemental Figure S1). With regard to specific molecular subclassifications, *HOXB5* and *HOXB7* are upregulated in the luminal A breast cancer cell lines, MCF-7 and ZR-75-1; *HOXB5*, *HOXB7* and *HOXB9* are upregulated in the TNBC cell lines, MDA-MB-231/MDA-MB-231-BR and BT-20; and *HOXB5* and *HOXB6* are upregulated in the HER2-enriched cell line, SK-BR-3, compared to MCF10a (Supplemental Figure S1). For *HOXC* cluster gene expression, the more posterior genes are highly expressed in MCF10a, with a similar pattern observed in the luminal A derived cell lines, MCF-7 and ZR-75-1. For TNBC, a shift in gene expression can be seen from posterior genes to anterior genes, when compared to MCF10a, with upregulated *HOXC4* and *HOXC5* expression in TNBC cell lines, whilst in the HER2-enriched cell line *HOXC* expression is generally upregulated, most notably *HOXC4* and *HOXC10*. *HOXD* genes are mostly not expressed in both MCF10a and breast cancer cell lines, except for *HOXD1* or *HOXD8* (Supplemental Figure S1).

For the TALE genes, high expression was seen across most TALE genes in MCF10a, particularly *PBX1-3* and *MEIS1*, whilst all breast cancer cell lines showed upregulated expression of only *PBX1*, with *PBX2*, *PBX3* and *MEIS1* exhibiting lower expression (Supplemental Figure S1).

### 3.2. Susceptibility of Breast Cancer Cell Lines to HTL-001-Induced Apoptosis

**Figure 2:**
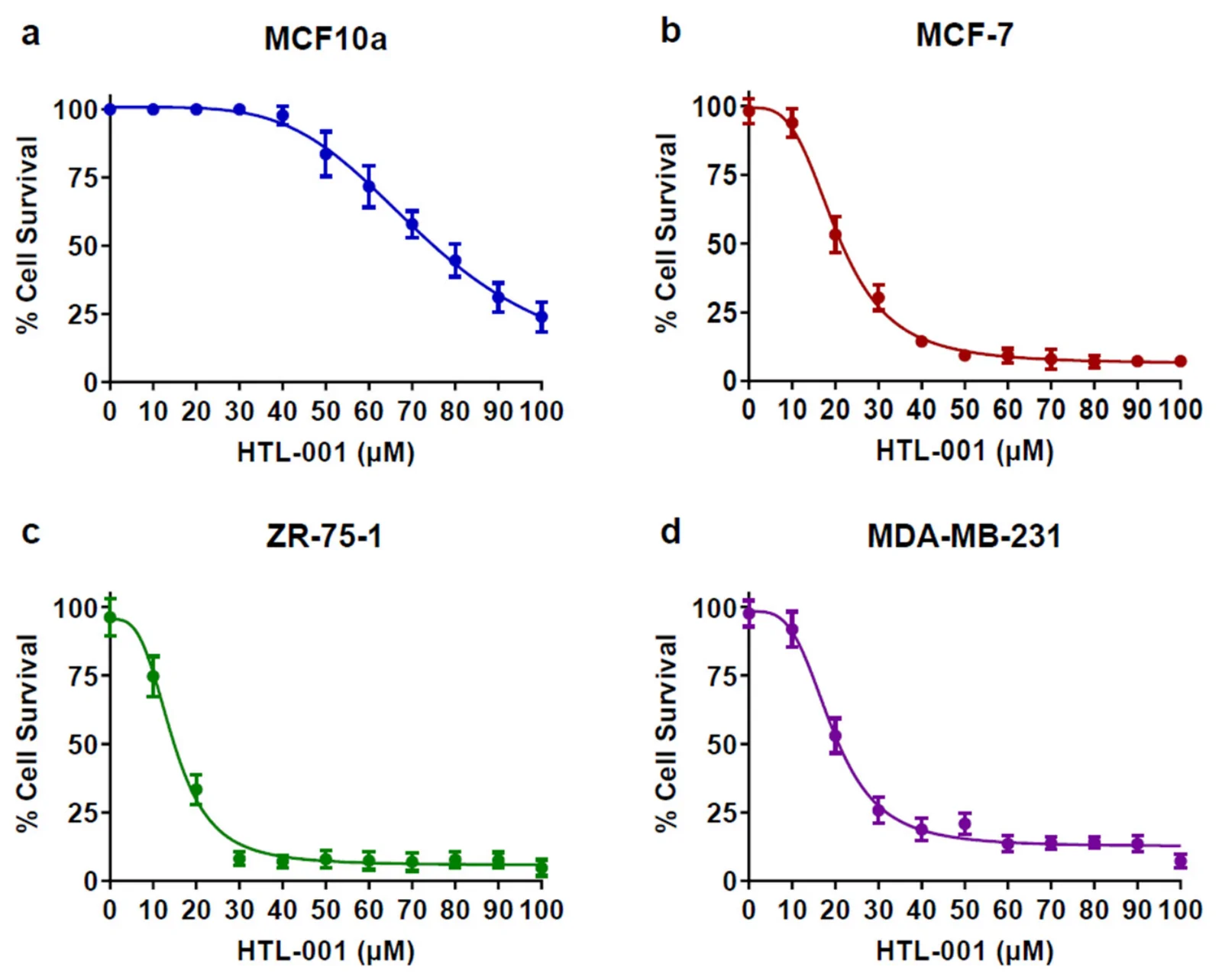

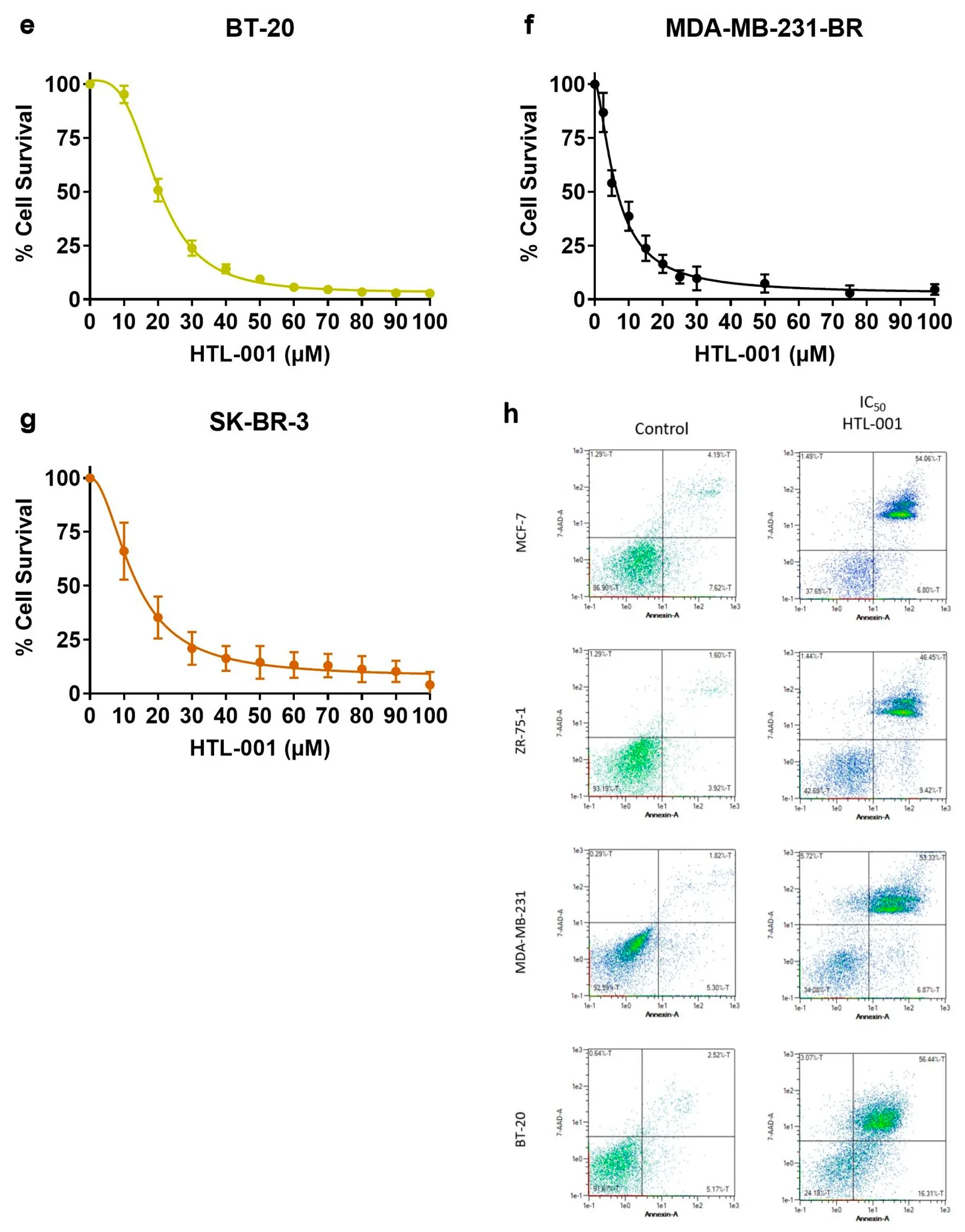
HTL-001 dose response curves for (**a**) MCF10a, a non-tumorigenic normal breast cell line (IC50 73 μM), (**b**) MCF-7, a luminal breast cancer cell line (IC50 21 μM), (**c**) ZR-75-1, a luminal breast cancer cell line (IC50 15 μM), (**d**) MDA-MB-231, a TNBC cell line (IC50 19 μM), (**e**) BT-20, a TNBC cell line (IC50 20 μM), (**f**) MDA-MB-231-BR, a TNBC cell line (IC50 6 μM), and (**g**) SK-BR-3, a HER2-enriched breast cancer cell line (IC50 13 μM). (**h**) Dual staining of Annexin and 7-AAD in MCF-7, ZR-75-1, MDA-MB-231 and BT-20, treated with the IC50 of HTL-001 vs. untreated cells, respective of each cell line; the colour represents increasing cell numbers red > green > blue. Cell survival has been normalised to 100% cell survival in untreated cells (media only). Each technical run was performed in quadruplet, and each biological experiment was performed in triplicate. Errors bars represent the standard deviation of the mean. Non-linear regression from a [inhibitor] vs. response–variable slope (four parameters) was used to calculate IC50 of HTL-001, respective of each cell line.

Given the high level of *HOX* gene dysregulation in the breast cancer-derived cell lines, we assessed the therapeutic potential of an antagonist of HOX-PBX interactions, HTL-001, in all the breast cancer cell lines and MCF10a. This revealed a dose-dependent cytotoxicity from HTL-001 treatment, with higher sensitivity in the breast cancer cell lines and lowest sensitivity in MCF10a, with an IC50 of 73 μM. Even at the highest dose of HTL-001, 100 μM, approximately 25% of MCF10a cells remained viable, whereas only 5–10% of breast cancer derived cells remained viable at the highest dose of HTL-001 (Figure 2). There was no statistical difference in sensitivity to HTL-001-induced cytotoxicity between different molecular subclassifications. MDA-MB-231-BR (TNBC) was the most sensitive to HTL-001, with an IC50 of 6 μM, whilst MCF-7 (luminal A) was the least sensitive to HTL-001 therapy, with an IC50 of 21 μM. The median IC50 for all the breast cancer cell lines was 16 μM. Dual staining with Annexin and 7-AAD confirmed the mode of cell death as late apoptosis in MCF-7, ZR-75-1, MDA-MB-231 and BT-20 cells treated with their respective IC50 of HTL-001, compared to untreated cells (Figure 2).

**Figure 3:**
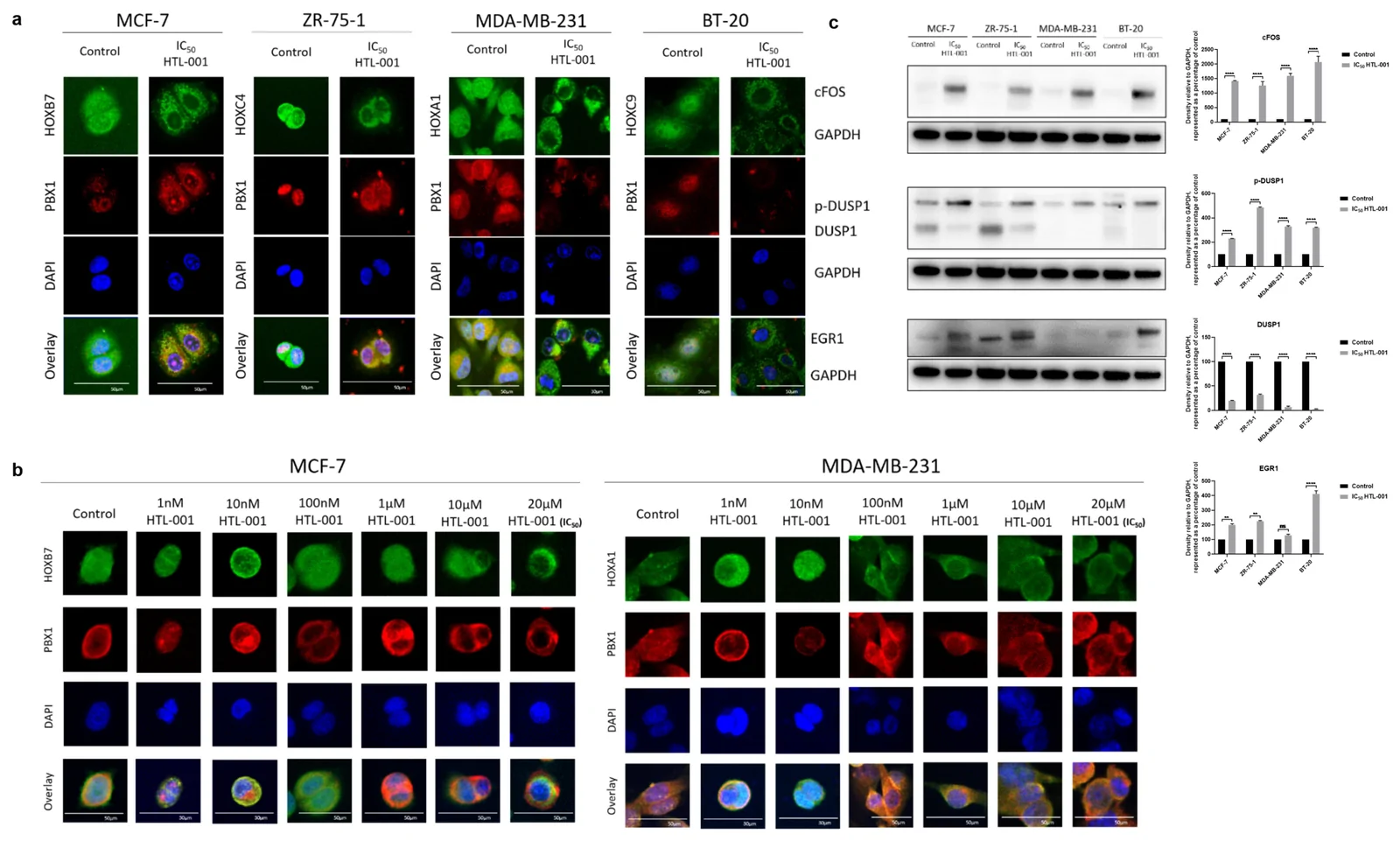
HTL-001 causes nuclear/cytoplasmic re-localisation of HOX and PBX proteins in breast cancer cell lines and increases the expression of pro-apoptotic proteins. (**a**) Cells were treated with media only or the IC50 of HTL-001, fixed, permeabilised and co-labelled with anti-PBX1 and either anti-HOXB7, anti-HOXC4, anti-HOXA1 or anti-HOXC9. HOX and PBX co-localisation was visualised by confocal immunofluorescence microscopy. (**b**) A time course experiment reveals that HTL-001 causes nuclear re-location of HOXA1, HOXB7 and PBX1 in MCF-7 and MDA-MB-231 cells within 10 min of HTL-001 treatment. (**c**) Western blot analysis and densitometry graphs of cFos, total DUSP1, phosphorylated DUSP1 (p-DUSP1) and EGR1 protein expression in breast cancer cells treated with HTL-001 vs. control cells. All breast cancer cell lines showed significant increase in cFOS, phosphorylated DUSP1 and EGR1 with a decline in total DUSP1 following HTL-001 treatment. GAPDH was used as a loading control and quantification of cFOS, DUSP1 and EGR1 expression was normalised to GAPDH expression. Each technical run was performed in duplicate and each biological experiment was performed in triplicate. Error bars represent the standard deviation of the mean. Statistical significance was determined by Kruskal–Wallis test. ns (*p* > 0.1234), ** (*p* < 0.0021), **** (*p* < 0.0001).

Previous studies have shown that disruption of HOX/PBX binding leads to the rapid re-localisation of HOX and PBX proteins from the nucleus to the cytoplasm, and a corresponding increase in pro-apoptotic proteins including phosphorylated DUSP1 (p-DUSP1), EGR1 and cFos [1]. In order to investigate whether a similar mechanism occurs in breast cancer-derived cell lines, we use used immunofluorescence microscopy to detect HOXA1, HOXB7, HOXC4, HOXC9 and PBX1 proteins in MCF-7, ZR-75-1, MDA-MB-231, and BT-20 cells before and after treatment with HTL-001 (Figure 3). This reveals a loss of each of these proteins from the nucleus in response to HTL-001, and a time course experiment in MCF-7 and MDA-MB-231 cells reveals that this occurs within 10 min of treatment (Figure 3). Western blotting also demonstrated an upregulation of p-DUSP1, EGR1 and cFos proteins after cells were treated with the IC50 concentration of HTL-001 peptide (Figure 3).

### 3.3. Sensitivity of Breast Cancer Cell Lines to HTL-001 Is Correlated to Dysregulated HOX and TALE Gene Expression

**Figure 4:**
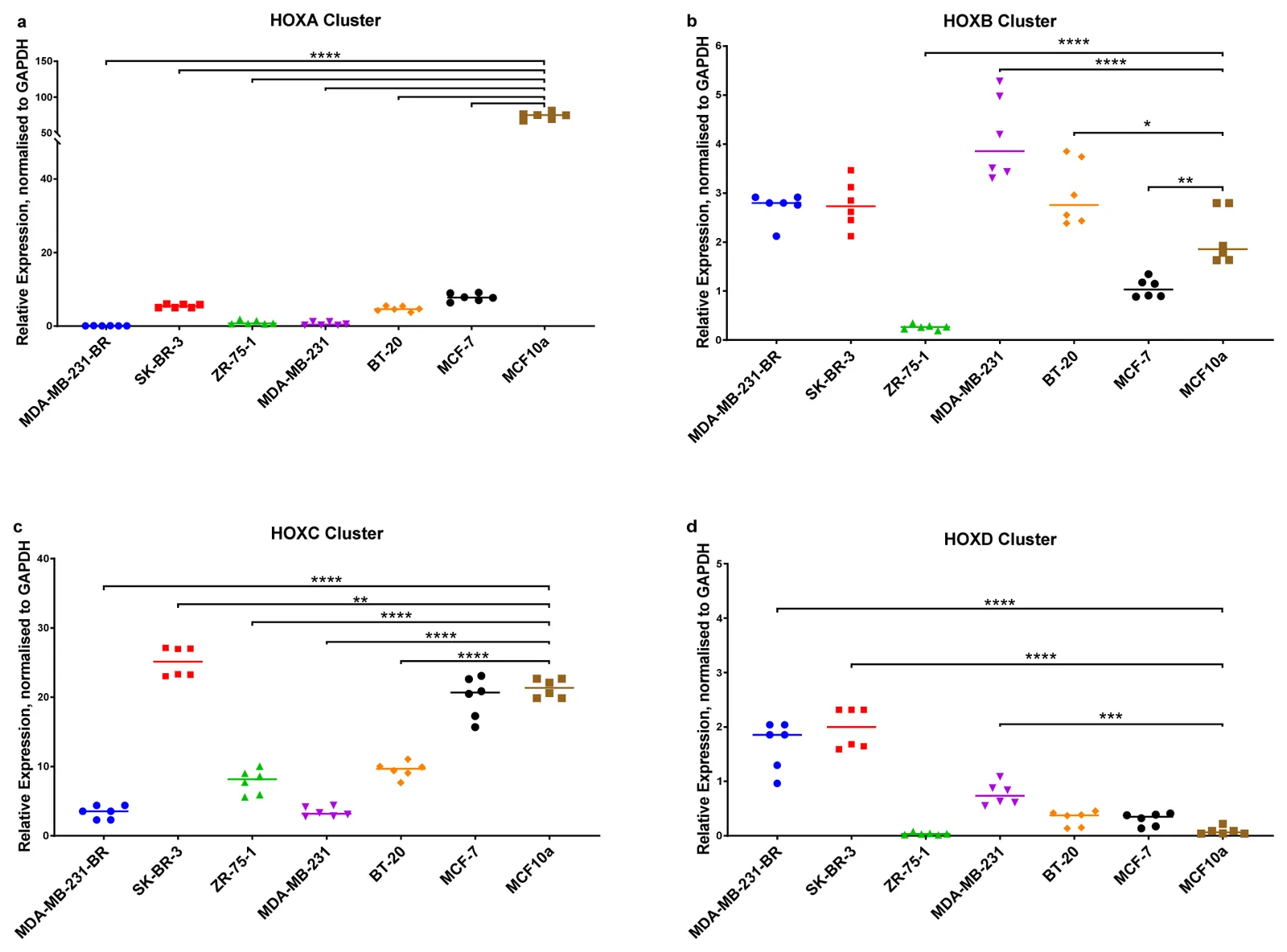

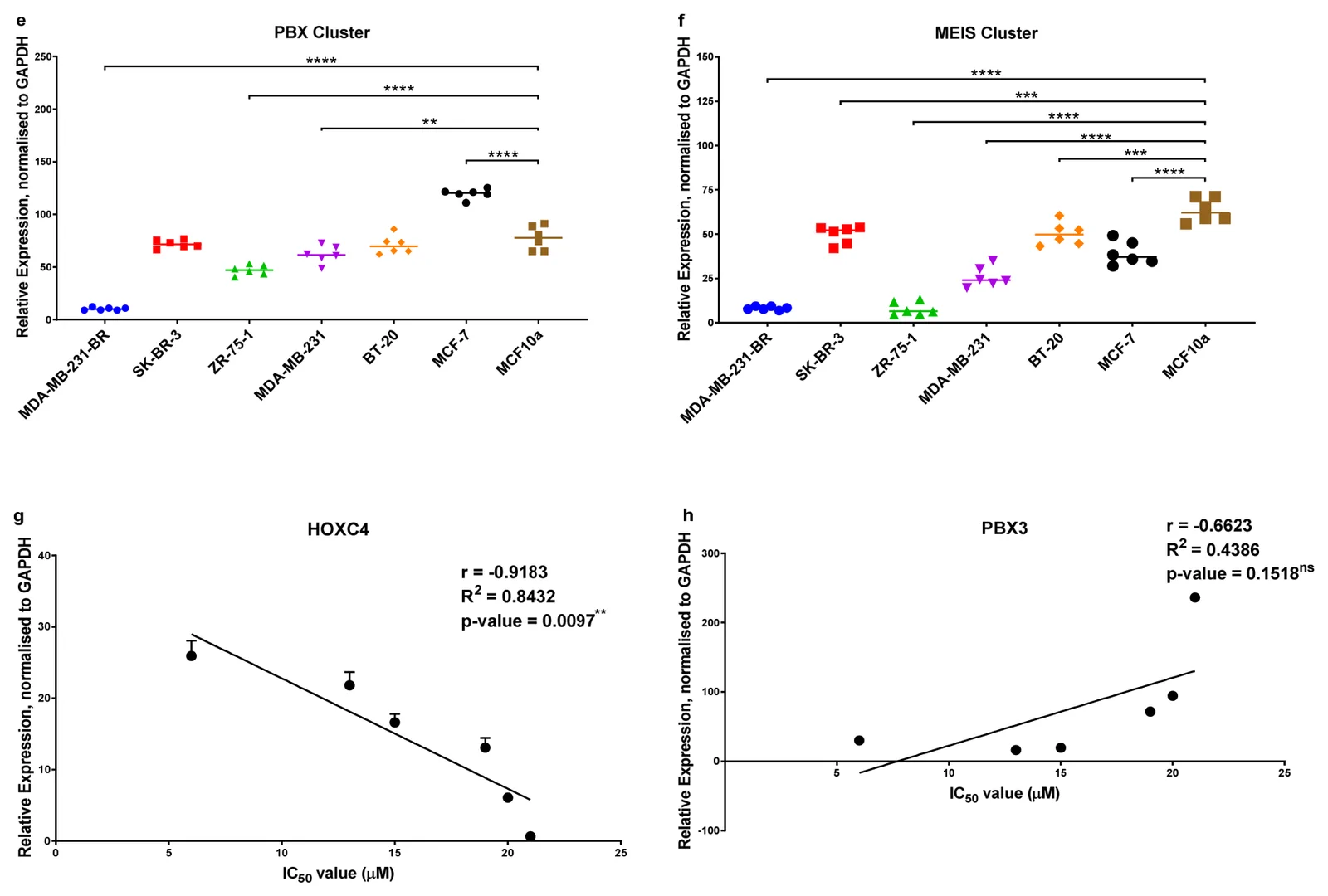
HTL-001 sensitivity correlates to the mean expression of specific *HOX* gene clusters and individual *HOX*, *PBX* and *MEIS* genes. Mean expression of *HOXA* (**a**), *HOXB* (**b**), *HOXC* (**c**), *HOXD* (**d**), *PBX* (**e**) and *MEIS* (**f**) cluster genes in cell lines listed in order of sensitivity is shown. There is an apparent linear regression of *HOXC4* (**g**) and *PBX3* (**h**) expression and IC50 of breast cancer cell lines. Statistical significance was determined by one-way ANOVA or Pearson’s Correlation. ns (*p* > 0.1234), * (*p* < 0.0332), ** (*p* < 0.0021), *** (*p* < 0.0002), **** (*p* < 0.0001).

Based on the *HOX*, *PBX* and *MEIS* expression data described above (Supplementary Figure S1), mean expression levels of genes in the different *HOX* clusters and the *PBX* and *MEIS* gene groups were calculated for each cell line and compared to HTL-001 sensitivity (Figure 4). Except for one cell line, a trend can be seen in the sensitivity to HTL-001-induced apoptosis and average gene expression of different subsets of these genes. Sensitivity to HTL-001-induced apoptosis is higher in cell lines with downregulated *HOXA* gene expression when the IC50 is less than 20 μM (Figure 4a), although there is no clear relationship between *HOXB* (Figure 4b), *HOXC* (Figure 4c) and *HOXD* (Figure 4d), *PBX* (Figure 4e) and *MEIS* (Figure 4f) expression and sensitivity.

When analysing the relationship between the expression of individual *HOX* and *PBX*/*MEIS* cofactor genes and sensitivity to HTL-001, only *HOXC4* (Figure 4g) and *PBX3* (Figure 4h) showed a significant correlation. Cell lines with higher *HOXC4* expression are more sensitive to HTL-001-induced apoptosis with the highest *HOXC4* expression and highest sensitivity to HTL-001 observed in MDA-MB-231-BR. Cell lines with lower *PBX3* expression were also more susceptible to HTL-001-induced cytotoxicity. *HOXC4* expression had the strongest relationship to HTL-001 sensitivity, with a negative correlation trend where r = −0.9183, whilst *PBX3* has a positive correlation to HTL-001 sensitivity and r value = 0.6623.

### 3.4. 5-aza and TSA Induce Both the Re-Expression of Silenced HOX Genes and the Silencing of Overexpressed HOX Genes

**Figure 5:**
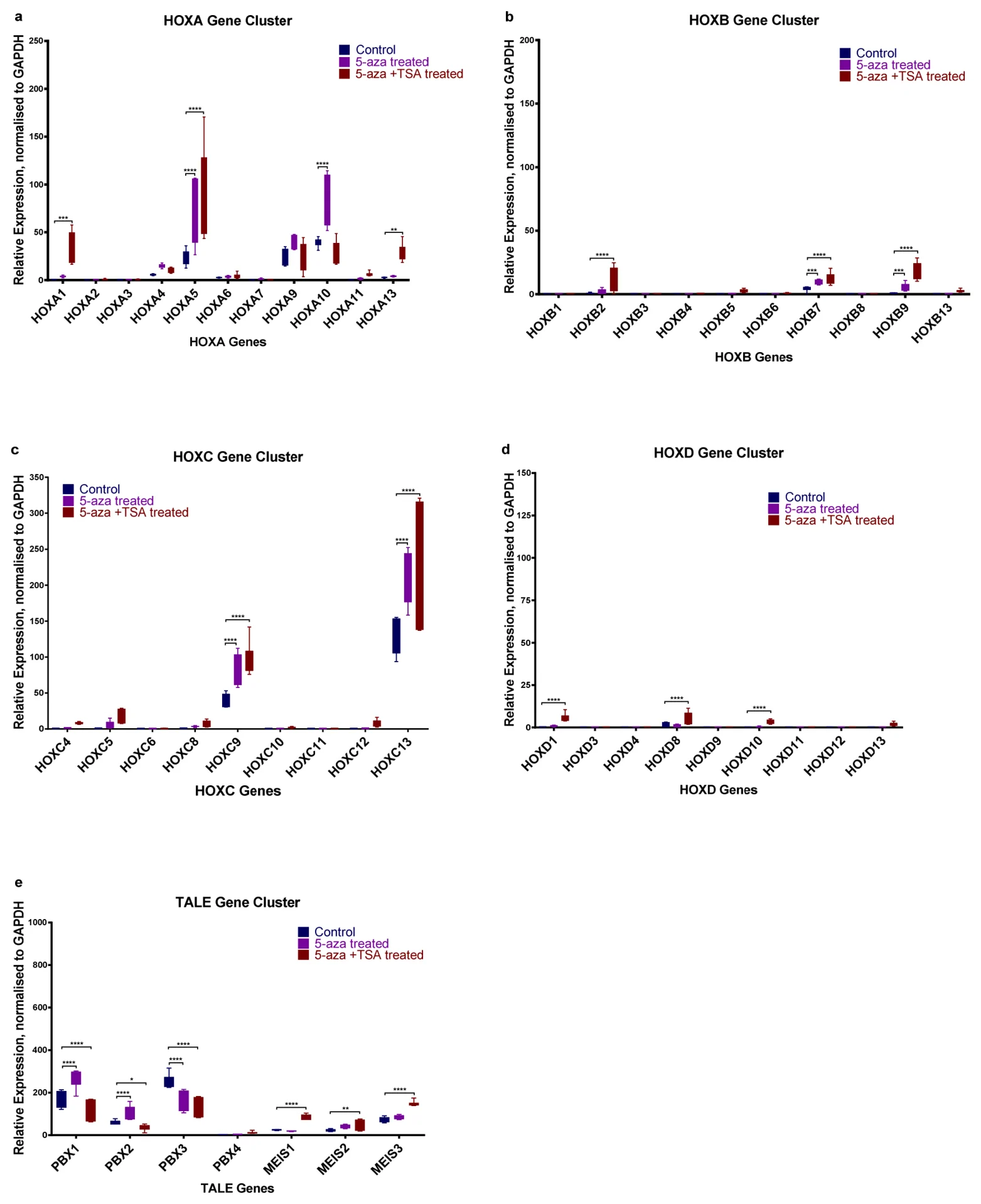
*HOX*, *PBX* and *MEIS* gene expression in MCF-7 cells in the presence of 5-aza with or without TSA for the (**a**) *HOXA* Cluster, (**b**) *HOXB* Cluster, (**c**) *HOXC* Cluster, (**d**) *HOXD* Cluster, and (**e**) *PBX*/*MEIS* genes. Gene expression has been normalised to the housekeeping gene, *GAPDH*, and multiplied by a factor of 10,000. Each technical run was performed in duplicate and each biological experiment was performed in triplicate. Error bars represent the standard deviation of the mean. Statistical significance was determined by Kruskal–Wallis test. * (*p* < 0.0332), ** (*p* < 0.0021), *** (*p* < 0.0002), **** (*p* < 0.0001).

**Figure 6:**
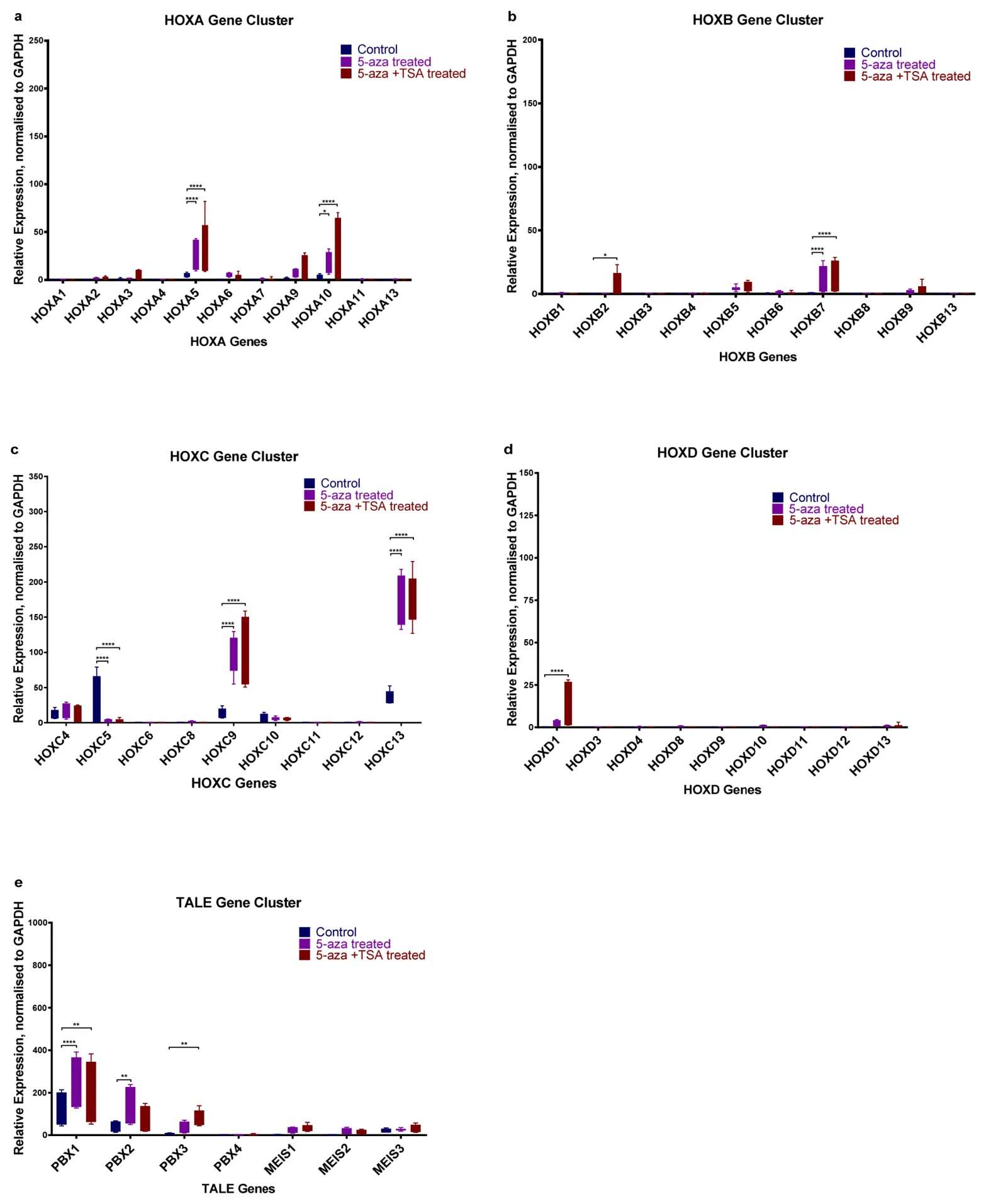
*HOX* and *TALE* (*PBX*/*MEIS*) gene expression in ZR-75-1 cells in the presence of 5-aza with or without TSA for the (**a**) *HOXA* Cluster, (**b**) *HOXB* Cluster, (**c**) *HOXC* Cluster, (**d**) *HOXD* Cluster, and (**e**) TALE Cluster. Gene expression has been normalised to the housekeeping gene, GAPDH, and multiplied by a factor of 10,000. Each technical run was performed in duplicate and each biological experiment was performed in triplicate. Error bars represent the standard deviation of the mean. Statistical significance was determined by Kruskal–Wallis test. * (*p* < 0.0332), ** (*p* < 0.0021), **** (*p* < 0.0001).

**Figure 7:**
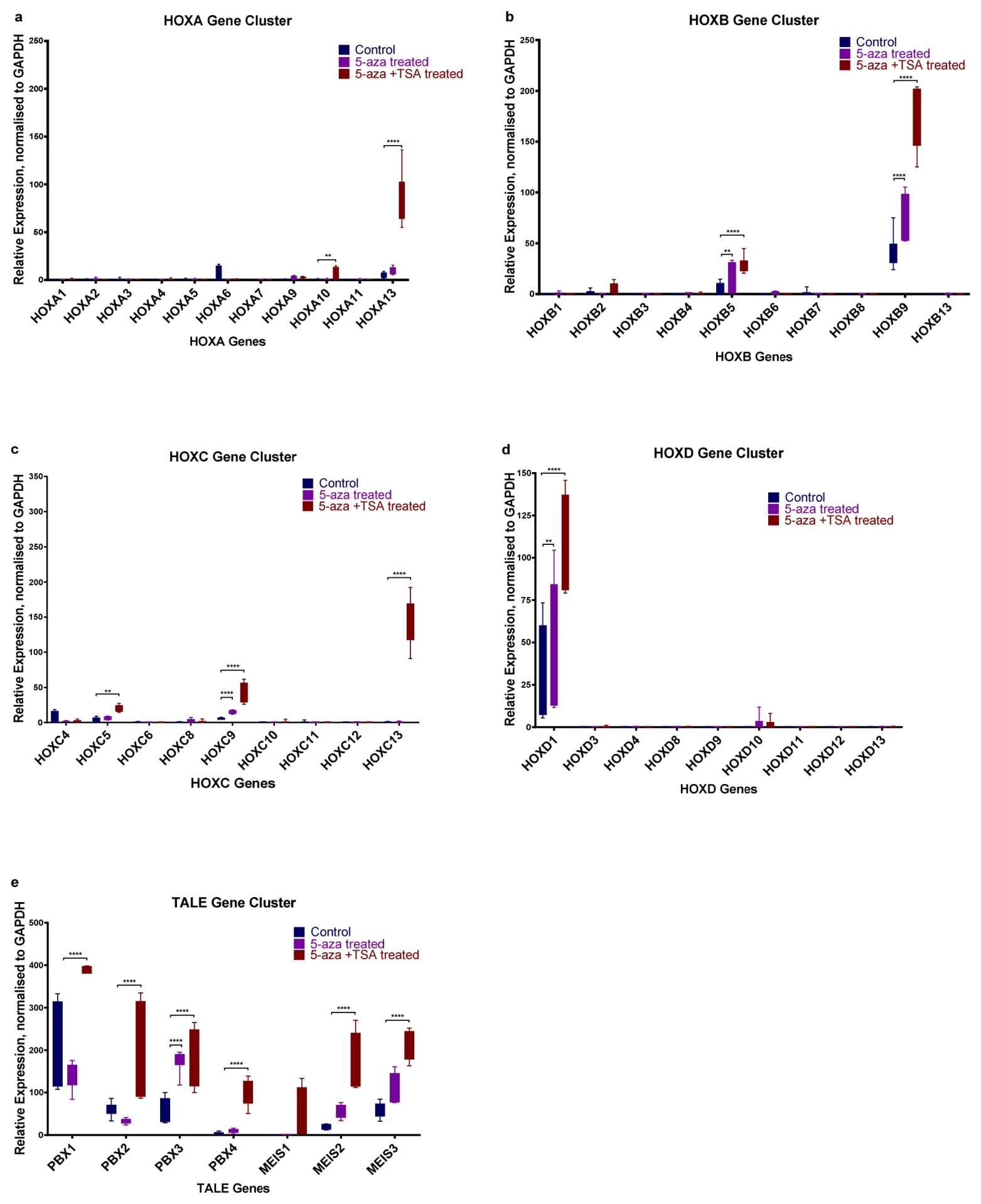
*HOX* and *TALE* (*PBX*/*MEIS*) gene expression in MDA-MB-231 cells in the presence of 5-aza with or without TSA for the (**a**) *HOXA* Cluster, (**b**) *HOXB* Cluster, (**c**) *HOXC* Cluster, (**d**) *HOXD* Cluster, and (**e**) *TALE* Cluster. Gene expression has been normalised to the housekeeping gene, *GAPDH* and multiplied by a factor of 10,000. Each technical run was performed in duplicate and each biological experiment was performed in triplicate. Error bars represent the standard deviation of the mean. Statistical significance was determined by Kruskal–Wallis test. ** (*p* < 0.0021), **** (*p* < 0.0001).

**Figure 8:**
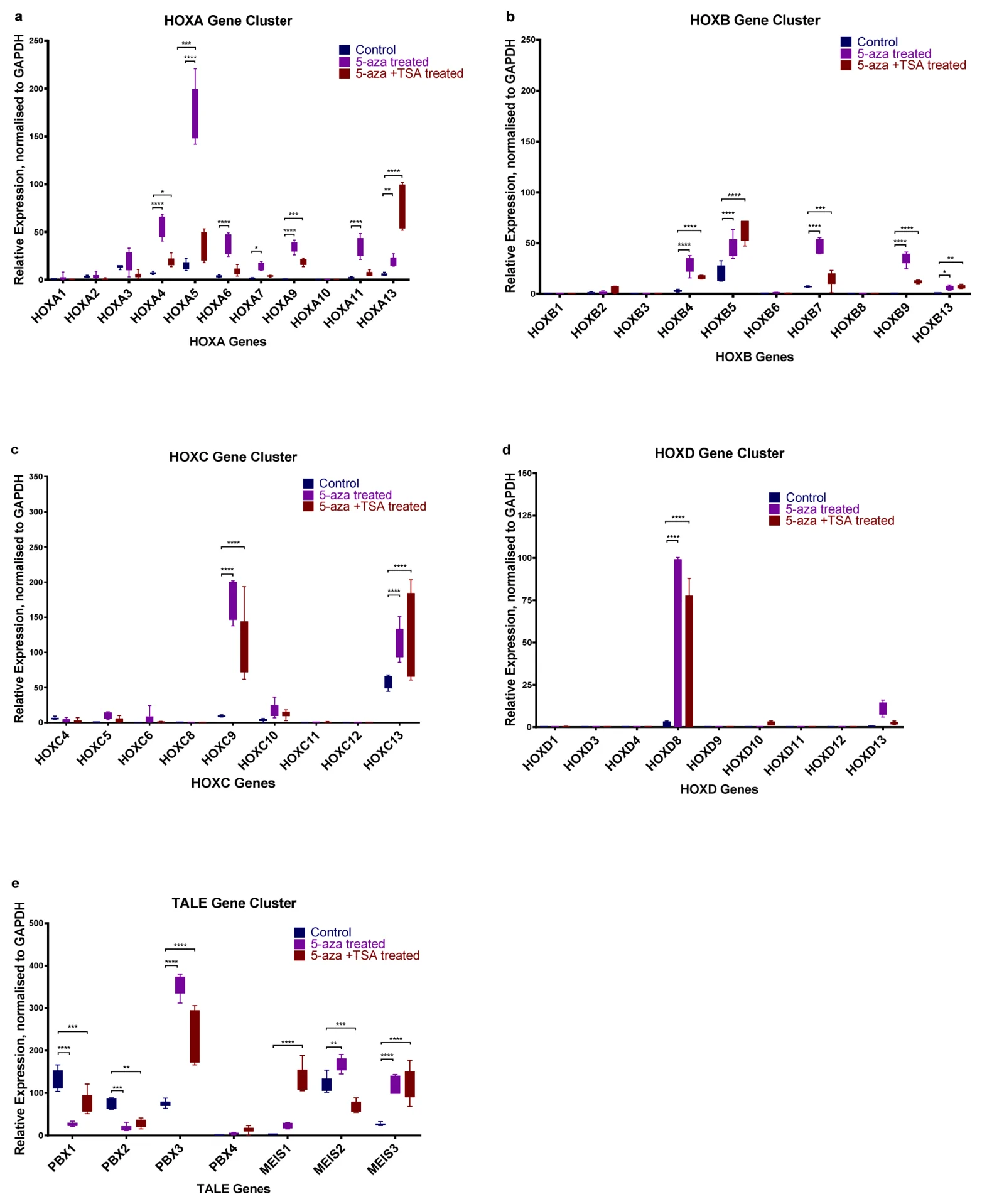
*HOX* and *TALE* (*PBX*/*MEIS*) gene expression in BT-20 cells in the presence of 5-aza with or without TSA for the (**a**) *HOXA* Cluster, (**b**) *HOXB* Cluster, (**c**) *HOXC* Cluster, (**d**) *HOXD* Cluster, and (**e**) *TALE* Cluster. Gene expression has been normalised to the housekeeping gene, *GAPDH*, and multiplied by a factor of 10,000. Each technical run was performed in duplicate and each biological experiment was performed in triplicate. Error bars represent the standard deviation of the mean. Statistical significance was determined by Kruskal–Wallis test. * (*p* < 0.0332), ** (*p* < 0.0021), *** (*p* < 0.0002), **** (*p* < 0.0001).

To evaluate whether epigenetic modifications might be responsible for *HOX*, *PBX*, and *MEIS* gene dysregulation, cells were incubated in 5-aza with/without TSA. Gene expression analysis of untreated and treated cells by RT-qPCR was used to analyse the expression of all 39 *HOX* genes and seven *PBX*/*MEIS* genes, in two luminal A breast cancer and two TNBC cell lines. Re-expression or silencing of *HOX* and *TALE* genes after 5-aza treatment or 5-aza and TSA treatment was assessed by comparative analysis against gene expression levels in untreated cells. This reveals silencing of a specific subset of *HOX*, *PBX*, and *MEIS* genes, and overexpression of a different subset after treatment (Figure 5, Figure 6, Figure 7 and Figure 8).

For the luminal A-derived MCF-7 cell line (Figure 5), a subset of silenced genes was re-expressed with 5-aza treatment and upregulated with the addition of TSA treatment including *HOXA1*, *HOXA5*, *HOXB2*, *HOXB7*, *HOXB9*, *HOXC5*, *HOXC9*, *HOXC13*, *HOXD1*, *MEIS2* and *MEIS3*. Some genes were only re-expressed in the presence of 5-aza alone including *HOXA4*, *HOXA9*, *HOXA10*, *PBX1* and *PBX2*, suggesting that they are silenced exclusively by DNA methylation, while others were only re-expressed with the addition of TSA, including *HOXA13*, *HOXC4*, *HOXD8* and *MEIS1*, indicating that they are mainly silenced by histone deacetylation. For the other luminal A-derived cell line, ZR-75-1 (Figure 6), the subset of silenced genes which were re-expressed with 5-aza treatment and additionally upregulated by TSA included *HOXA5*, *HOXA9*, *HOXA10*, *HOXB5*, *HOXB7*, *HOXC9*, *HOXC13*, *PBX3* and *MEIS1*. *HOXA6*, *PBX1* and *PBX2* were only re-expressed in the presence of 5-aza, whilst *HOXB2* and *HOXD1* were the only genes silenced by the addition of TSA only.

For both these cell lines, most genes in the *HOXA* and *HOXB* gene clusters were re-expressed with 5-aza and transcriptionally reactivated with TSA. *PBX1* and *PBX2* were re-expressed with 5-aza only in both cell lines, indicating some scope of similar epigenetically modified profiles in luminal A breast cancer.

Clearer trends were apparent for the TNBC cell lines, MDA-MB-231 and BT-20. In MDA-MB-231 cells (Figure 7), 5-aza and TSA treatment resulted in the re-expression of several silenced genes including *HOXA13*, *HOXB5*, *HOXB9*, *HOXC9*, *HOXD1*, *PBX3*, *MEIS2* and *MEIS3*. Genes that were exclusively re-expressed with the addition of TSA were *HOXA10*, *HOXB2*, *HOXC5*, *HOXC13*, *PBX1*, *PBX2*, *PBX4* and *MEIS1*. In BT-20, genes that were re-expressed after 5-aza and TSA treatment included *HOXA13*, *HOXB5*, *HOXB13*, *HOXC13*, *HOXD8* and *MEIS3*. A large subset of genes in BT-20 were re-expressed with 5-aza treatment alone including *HOXA3*, *HOXA4*, *HOXA5*, *HOXA6*, *HOXA7*, *HOXA9*, *HOXA11*, *HOXB4*, *HOXB7*, *HOXB9*, *HOXC9*, *HOXC10*, *HOXD13*, *PBX3* and *MEIS2* (Figure 8). Other genes which were only re-expressed with the addition of TSA included *HOXB2*, *PBX1*, *PBX2* and *MEIS1*. Comparing these two cell lines suggests that a few genes in the *HOXB* gene cluster are hypermethylated and silenced. Within both cell lines, *HOXB2*, *PBX1*, *PBX2* and *MEIS2* were only re-expressed with the inhibition of histone deacetylation.

### 3.5. 5-aza and TSA Treatment Generally Reduces the Sensitivity of Breast Cancer Cell Lines to HTL-001

We examined the sensitivity of the same four cells lines to HTL-001 after 5-aza with or without TSA. Cells were subjected to demethylating conditions as described above and then treated with varying concentrations of HTL-001 within a range of 0 to 100 μM for 2 h. Dose response curves were generated for all subsets of cell populations as shown in Supplementary Figure S2. All four cell lines show a difference in sensitivity to HTL-001-induced apoptosis after 5-aza +− TSA treatment. The IC50 values are summarised in Table 1. After 5-aza treatment, the sensitivity of MCF-7 cells to HTL-001-induced apoptosis is increased, and further heightened when cells are treated with 5-aza and TSA. The opposite effect is seen in ZR-75-1 and MDA-MB-231 cells, and for BT-20 cells HTL-001-induced apoptosis is increased with 5-aza treatment alone.

### 3.6. HTL-001 Acts Synergistically with Chemotherapeutic Agents at Low Doses

In order to assess possible synergistic relationships between HOX/PBX inhibition through HTL-001 and chemotherapeutic agents, we examined the interaction between HTL-001 and Paclitaxel, 5-FU, Palbociclib, and Metformin. All these drugs show activity against the TNBC cell lines used in this study (Supplementary Figure S3).

**Figure 9:**
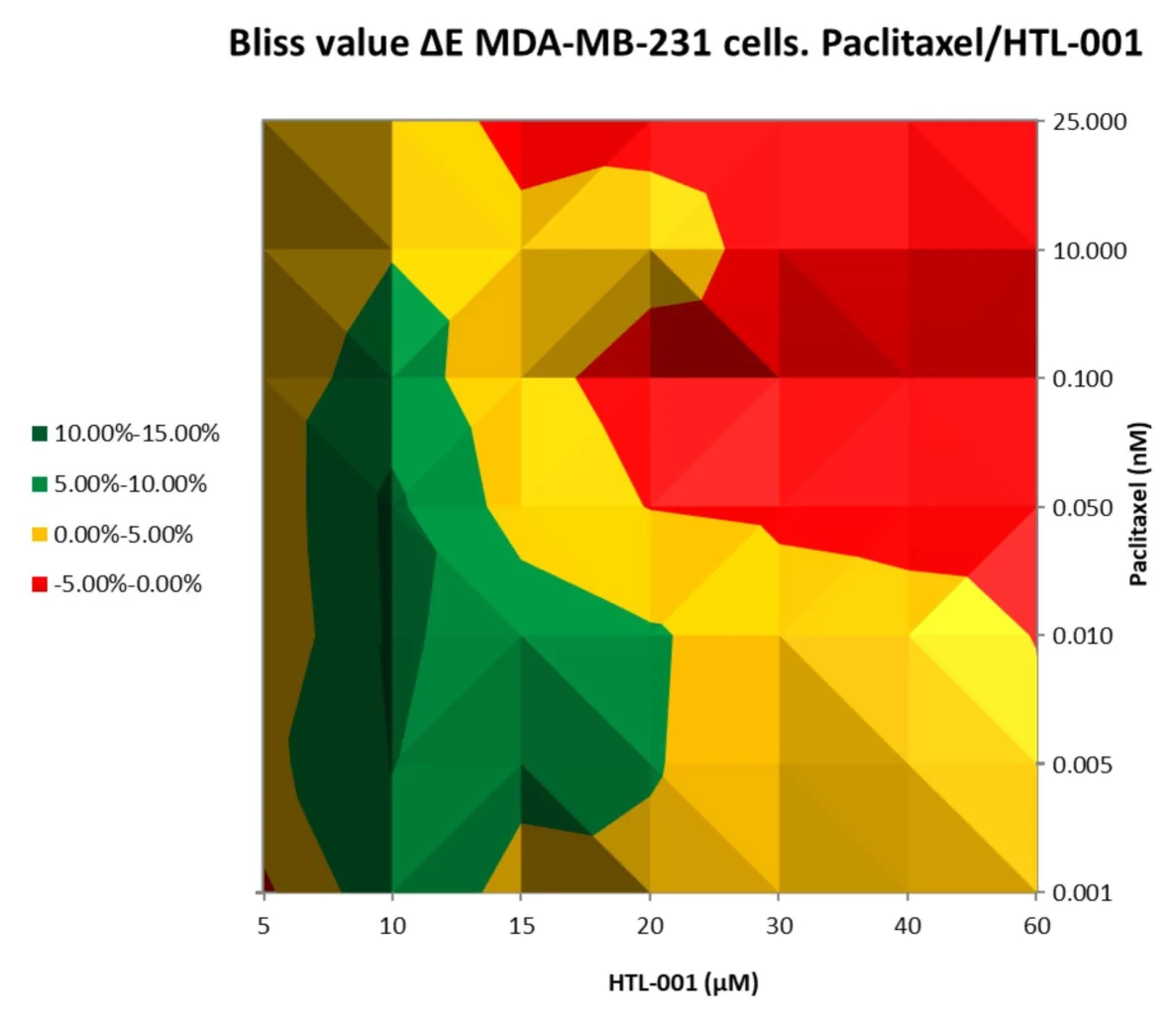

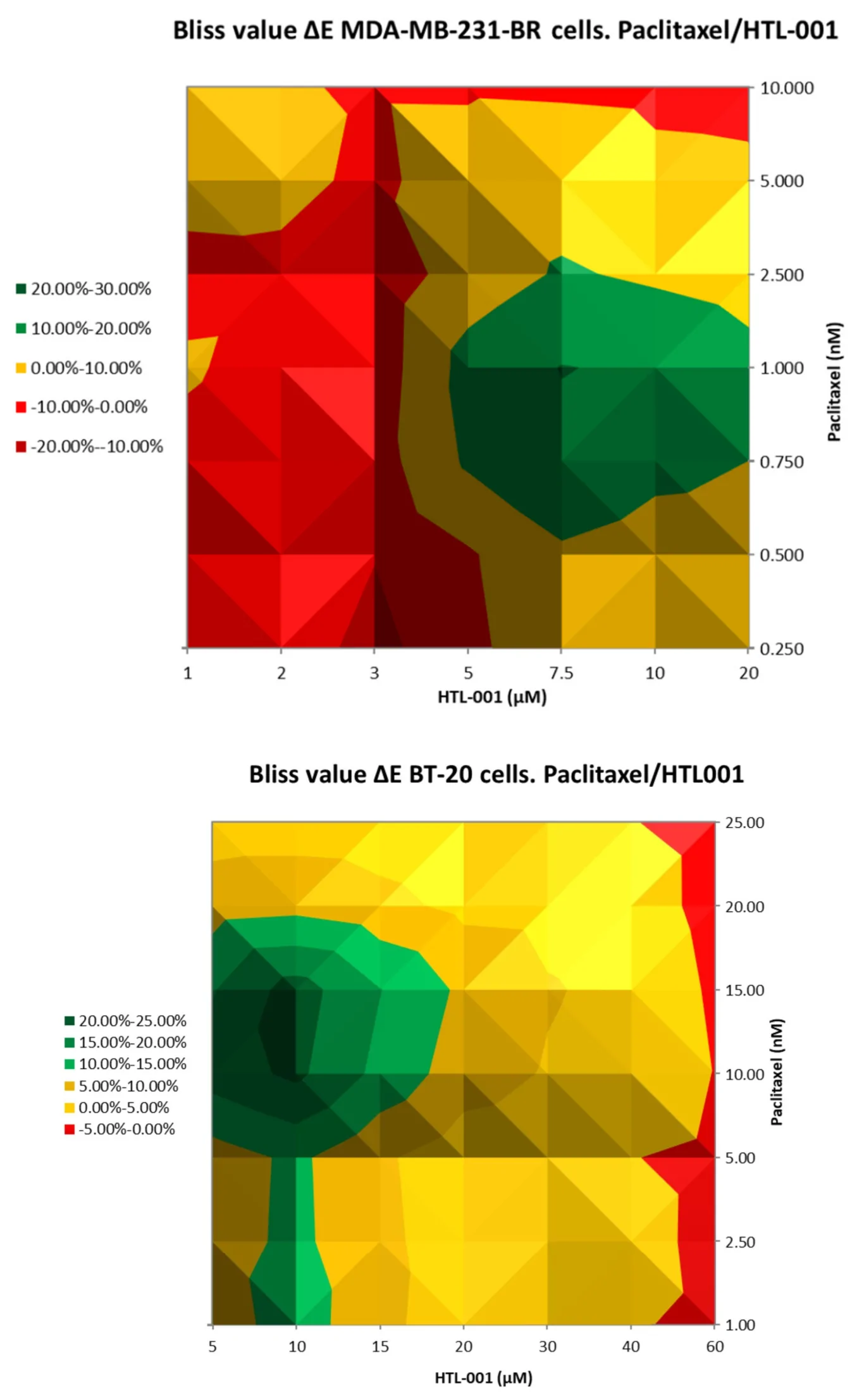
The averaged ΔE values for each combinational treatment with HTL-001 and Paclitaxel in MDA-MB-231, MDA-MB-231-BR, and BT-20 cells are plotted into a contour map to visualise the level of interaction. Colour coding represents the type of interaction and the extent of this interaction. Green represents positive ΔE values and indicates synergism, red represents negative ΔE values and indicates antagonism, and yellow represents neutral ΔE values and indicates Bliss Independence and additivity. Each technical run was performed in triplicate, and each biological experiment was performed in triplicate. The Bliss Independence Model was used to calculate ΔE values for each synergistic combinational treatment.

MDA-MB-231, MDA-MB-231-BR, and BT-20 cell lines were treated with HTL-001 or Paclitaxel alone, and in combination with each other across a range of doses, and cell survival was measured after 48 h. The interaction between these drugs was evaluated by Bliss Independence Analysis (Figure 9). For both cell types, synergy was only seen with doses of HTL-001 lower than the IC50 (approximately 20 μM for both cell lines). Similarly, synergy could only be seen with doses of Paclitaxel lower than the IC50 (0.16 nM for MDA-MB-231 and 28 nM for BT-20). Pockets of extremely high synergistic effect can be seen at very specific combined doses of HTL-001 and Paclitaxel but for both cell lines was seen with 10 μM HTL-001. The combination with the highest synergistic anti-cancer effect with the maximum percentage of cell death in BT-20 cells was 10 μM HTL-001 with 15 nM Paclitaxel. Antagonist effects of combining HTL-001 and Paclitaxel were only seen at the highest doses of HTL-001 and Paclitaxel, especially ones higher than their respective IC50 values. A highly synergistic pocket is induced when HTL-001 and Paclitaxel is combined in MDA-MB-231-BR cells (Figure 9). However, unlike in MDA-MB-231 and BT-20, this exists at doses of HTL-001 at the IC50 or slightly above (although at a similar dose to that needed for synergy in the other two cell lines).

**Figure 10:**
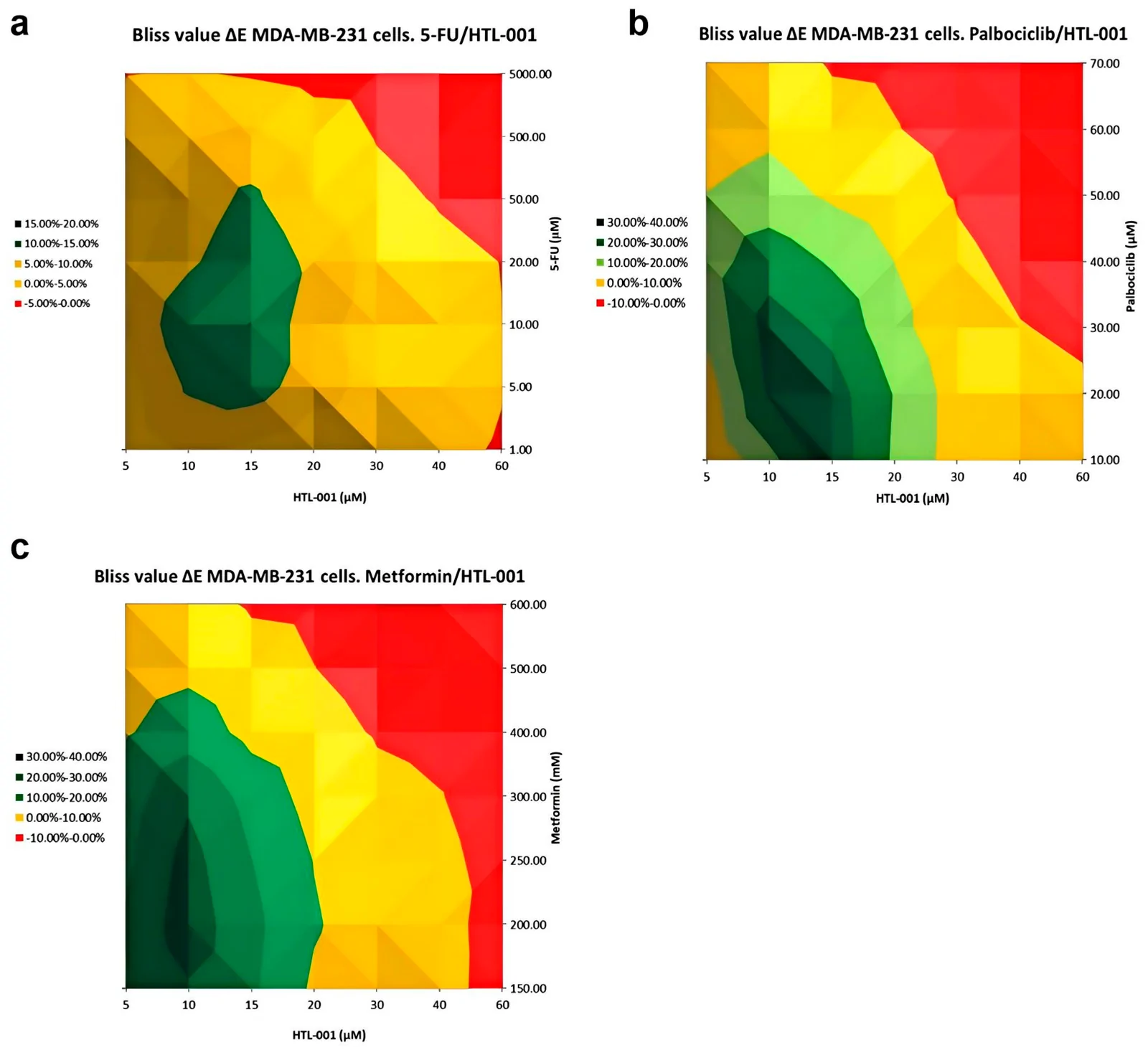
The averaged ΔE values for each synergistic combinational treatment in MDA-MB-231 cells are plotted into contour maps to visualise the level of interaction between HTL-001 and (**a**) 5-FU, (**b**) Palbociclib, and (**c**) Metformin. Colour coding represents the type of interaction and the extent of this interaction. Green represents positive ΔE values and indicates synergism, red represents negative ΔE values and indicates antagonism, and yellow represents neutral ΔE values and indicates Bliss Independence and additivity. Each technical run was performed in triplicate, and each biological experiment was performed in triplicate. Bliss Independence Model was used to calculate ΔE values for each synergistic combinational treatment.

Similar findings were also made for HTL-001 and either 5-FU, Palbociclib or Metformin in MDA-MB-231 (Figure 10), with a very specific synergy pocket at doses lower than the IC50 of each drug.

### 3.7. In Vivo Administration of HTL-001 Alone or in Combination with Paclitaxel Significantly Reduced MDA-MB-231-BR Tumour Growth

The ability of MDA-MB-231-BR cells to readily metastasize to the brain and their origin from a TNBC means that it reflects one of the clinically most aggressive forms of the disease. Furthermore, these cells are the most sensitive to killing by HTL-001 from the cell lines tested in this study, and the in vitro synergy studies described above indicate that there is very little risk of an antagonistic interaction between HTL-001 and Paclitaxel except at the very highest doses of the latter. We therefore selected this combination to test in vivo.

**Figure 11:**
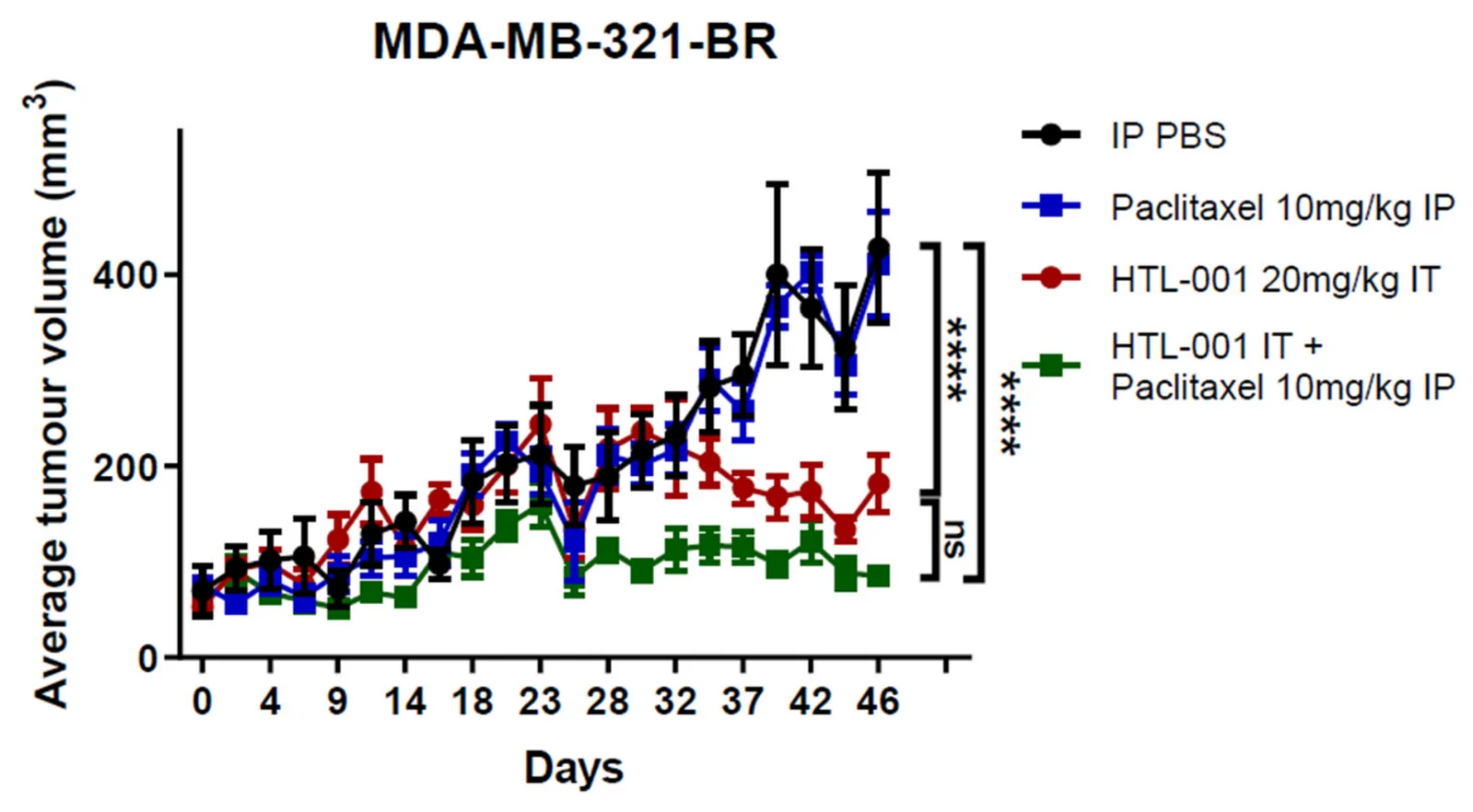
HTL-001 reduces tumour growth in vivo. Female BALBc nude mice were inoculated with 5 × 10^6^ MDA-MB-231-BR cells; *n* = 8 in each group. When the tumours reached 50–60 mm^3^, mice were dosed with either PBS, Paclitaxel alone, HTL-001 alone or Paclitaxel and HTL-001 in combination for five consecutive weeks. HTL-001 alone or in combination with Paclitaxel resulted in significant tumour control compared to Paclitaxel or PBS alone. Error bars show the standard error from the mean volume. Statistical significance was determined by two-way ANOVA. ns (*p* > 0.1234), **** (*p* < 0.0001).

A sub-therapeutic dose of 10 mg/kg was used for Paclitaxel and the standard tolerable dose of 20 mg/kg was used for HTL-001. As seen in Figure 11, HTL-001 alone or in combination with Paclitaxel resulted in significant tumour control compared to Paclitaxel or PBS alone (*p* < 0.0001), although we note that the combination of intratumoral and intraperitoneal injections only in the Paclitaxel plus HTL-001 group is a potential confounding factor. HTL-001 in combination with Paclitaxel resulted in a smaller final tumour size compared to HTL-001 alone, although this difference was not statistically significant (*p* = 0.0628).

## 4. Discussion

Advanced refractory breast cancer remains a highly prevalent and significant health issue for women, associated with reduced quality and quantity of life. *HOX* gene dysregulation has been shown to have a potentially key role in the development and progression of breast cancer, and HOX gene products are therefore potential therapeutic targets. In this study we have analysed the expression of *HOX* and *TALE* genes in cell lines derived from different molecular subtypes of breast cancer, as well as MCF10a which is derived from non-tumorigenic normal breast cells. Whilst there are significant variations in expression between the cell lines, a common observation for the malignant lines is a loss of expression of *HOXA* cluster genes. This reflects previous studies in which *HOXA* expression was shown to be repressed in breast tumours through changes in methylation and histone modifications throughout the 100 kb region of the cluster, but not beyond it [17]. A corresponding overexpression of Polycomb genes has been shown in both atypical ductal hyperplasia (ADH) and ductal carcinoma in situ (DCIS) precancerous lesions, which might drive these epigenetic changes [18]. Our findings reflect this, as we observed significant increases in *HOXA* gene expression when cells are treated with 5-aza that reverses methylation and TSA, which blocks histone modifications. This is accompanied to a lesser extent by increases in individual *HOX* genes from the other clusters and several of the *TALE* genes.

In addition to the *HOXA* genes, the 5′ members of the *HOXB* cluster also show a high level of dysregulation in breast cancer cell lines, with very high expression compared to MCF10a cells, most notably *HOXB5* and *HOXB7*. Both of these genes have been shown to have pro-oncogenic roles in breast cancer. *HOXB5* has been shown to promote breast cancer progression through increased invasion and migration via activation of the wnt/beta-catenin signalling pathway [19] and direct transcriptional activation of EGFR [20]. Its increased expression is also associated with resistance to Tamoxifen [21].

Inhibition of the interaction between HOX and PBX was previously shown to cause apoptosis in breast cancer-derived cell lines [12], and, similarly, inhibiting HOX/PBX binding in this study using HTL-001 caused cell death in a dose-dependent manner, with the cancer-derived lines being more sensitive than MCF10a. This sensitivity was found to have a significant negative correlation with the expression of *HOXC4* (r = −0.9183 *p* = 0.0097), which indicates that *HOXC4* might have a particularly significant role in breast cancer in blocking apoptosis through the activation of Fos, DUSP1 and FasL, previously identified as mediators of cell death following HOX/PBX inhibition [12]. This is supported by a previous study in which *HOXC4* was shown to facilitate breast cancer development by increasing signalling through the TGF-b/SMAD pathway [22], which is otherwise repressed by HOX/PBX inhibition through an increase in DUSP1 expression [1]. Except for MCF-7, the sensitivity of the breast cancer cell lines to HTL-001 decreases after treatment with 5-aza and TSA that block DNA methylation and reverse histone modifications, respectively (Table 1), which might be explained by the resulting generalised increase in *HOX* and *PBX* expression.

In contrast to these findings with 5-aza and TSA, we demonstrated synergistic interactions between HTL-001 and several commonly used chemotherapeutics including Paclitaxel, 5-FU, and Palbociclib, with an optimal HTL-001 concentration of around 10 μM for all the cell lines and drugs tested (Figure 9 and Figure 10). Tubulin inhibitors such as Paclitaxel have been shown to cause a general reduction in HOX expression [23], which might contribute to its synergistic interaction with HTL-001, although the pro-apoptotic effects of disrupting HOX/PBX dimers might also play a role [12]. 5-FU exerts a cytotoxic activity primarily through disrupting translation as a result of the incorporation of its metabolites into RNA. There have been no studies, as far as we are aware, that have directly examined the role of HOX and PBX dimers in translation. However, HOXA9 has been shown to directly interact with eukaryotic translation initiation factor 4E (eIF4E) and thereby enhance its activity [24]. In addition, inhibition of CDK4 and CDK6 by Palbociclib in acute myeloid leukaemia (AML) has been shown to cause apoptosis through a mechanism that involves a reduction in HOXA9 expression [25], which again suggests a possible mechanistic basis for a synergistic interaction with HTL-001.

HTL-001 administration in mice bearing MDA-MB-231-BR tumours caused a significant reduction in tumour growth compared to mice administered with PBS alone (Figure 11), comparable to results obtained using the HXR9-mediated HOX/PBX inhibitor in a mouse model of breast cancer [12]. Co-administration of HTL-001 and 10 mg/Kg Paclitaxel resulted in a further reduction in average tumour size between 28 and 46 days, although this difference was not statistically significant.

In summary, our study highlights the complex and subtype-specific dysregulation of HOX and TALE genes in breast cancer, most notably the repression of the HOXA cluster and overexpression of oncogenic HOXB members. The restoration of HOX gene expression through epigenetic modifiers such as 5-aza and TSA underscores the role of methylation and histone modifications in silencing these genes. Disruption of HOX/PBX interactions in vitro using HTL-001 induces apoptosis and is synergistic with several established chemotherapeutics including Paclitaxel and epigenetic modifiers, and in vivo HTL-001 can retard the growth of a TNBC tumour model, providing a rationale for further evaluation of HOX/PBX-targeted interventions.

## Figures and Tables

**Table 1:** IC50 values for each treatment in cell lines. Statistical significance was determined by two-way ANOVA. * (*p* < 0.0332), ** (*p* < 0.0021), *** (*p* < 0.0002).

Cell Line			HTL-001 IC_50_ Value (μM)	
	Control	5-aza-Treated	5-aza + TSA-Treated	*p*-Value (Control vs. 5-aza + TSA-Treated)
MCF-7	23.63	20.94	17.85	0.0018 **
ZR-75-1	13.77	21.14	26.61	<0.0001 ***
MDA-MB-231	17.14	17.83	17.9	<0.0001 ***
BT-20	21.16	37.35	18.32	0.0187 *

## Data Availability

The original contributions presented in this study are included in the article/Supplementary Material. Further inquiries can be directed to the corresponding author.

## Supplementary Materials

The following supporting information can be downloaded at: https://www.mdpi.com/article/10.3390/cimb48080800/s1.

## Abbreviations

5-aza, 5-azacytidine; 5-FU, 5-Fluorouracil; ADH, Atypical ductal hyperplasia; AML, Acute myeloid leukaemia; ANOVA, Analysis of variance; ATCC, American Type Culture Collection; DCIS, Ductal carcinoma in situ; EGF, Epidermal Growth Factor; EGFR, Epidermal Growth Factor Receptor; eIF4E, Eukaryotic translation initiation factor 4E; EMT, Epithelial to mesenchymal transition; FBS, Foetal Bovine Serum; FGF2, Fibroblast Growth Factor 2; GAPDH, Glyceraldehyde-3-Phosphate Dehydrogenase; MEIS, Myeloid Ecotropic Viral Integration Site 1 Homologue; PBX, Pre-B-cell Leukaemia Homeobox; STR, Short tandem repeat; TALE, Three Amino Acid Loop Extension; TNBC, Triple negative breast cancer; TSA, Trichostatin A.
