# Supplementary material for: Inhibition of HOX/PBX Dimers as a Potential Therapeutic Strategy in Breast Cancer Subtypes Including Triple Negative Breast Cancer
Source: Curr Issues Mol Biol. 2026 Aug 7;48(8):800. doi: 10.3390/cimb48080800 (PMC13511161; doi:10.3390/cimb48080800)
Supplement: Supplementary file 1 [file cimb-48-00800-s001.zip › cimb-4359450-supplementary.pdf]

Supplemental figures

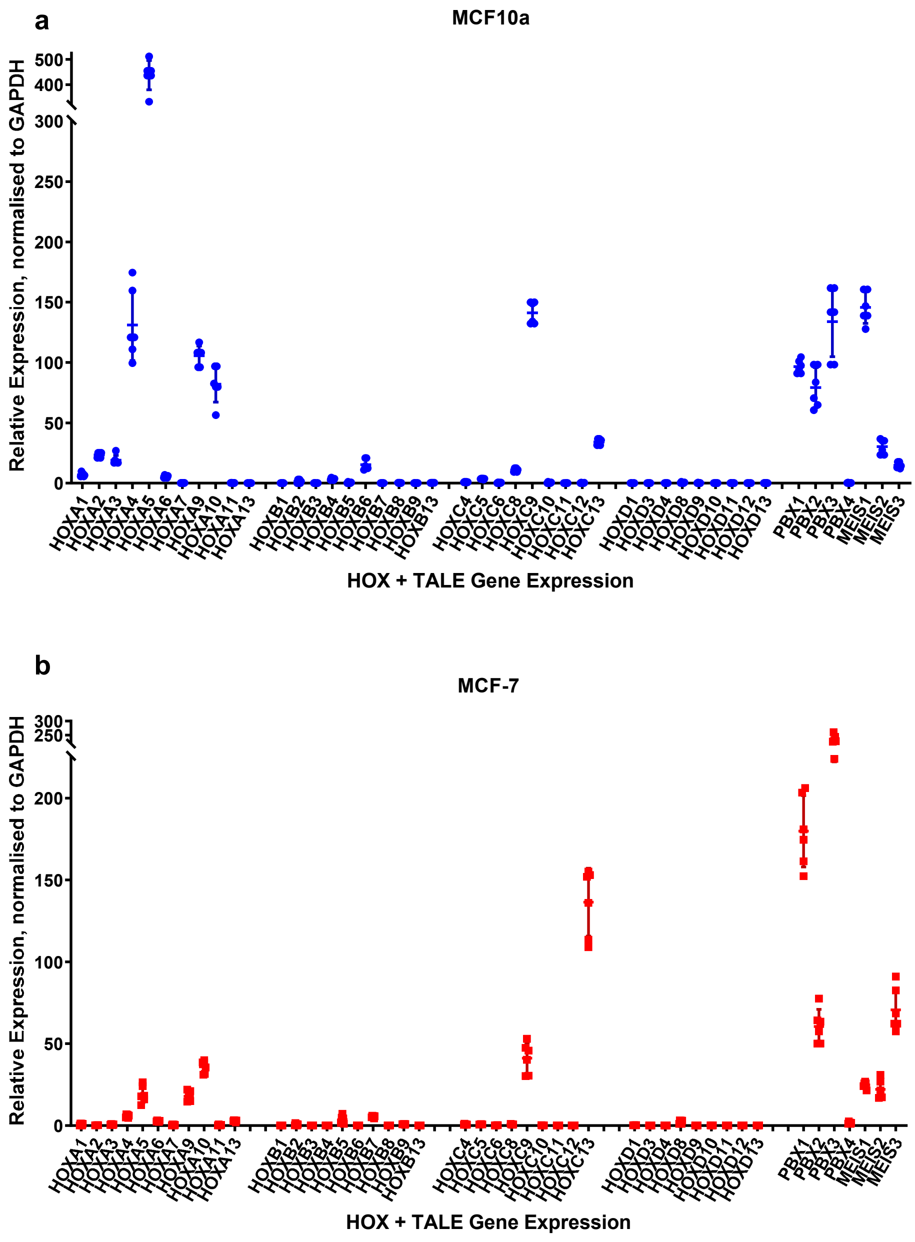

c

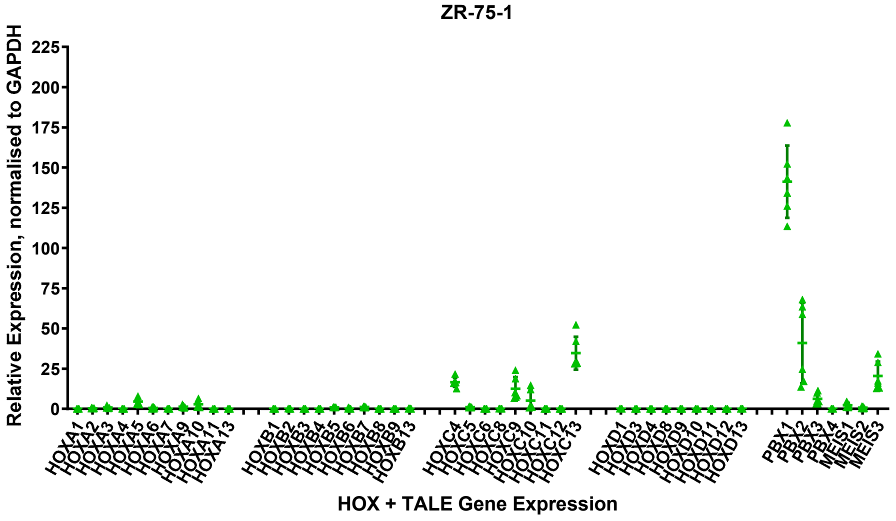
ZR-75-1
Relative Expression, normalised to GAPDH
225
200
175
150
125
100
75
50
25
0
HOXA1 HOXA2 HOXA3 HOXA4 HOXA5 HOXA6 HOXA7 HOXA9 HOXA10 HOXA11 HOXA13
HOXB1 HOXB2 HOXB3 HOXB4 HOXB5 HOXB6 HOXB7 HOXB8 HOXB9 HOXB13
HOXC4 HOXC5 HOXC6 HOXC8 HOXC9 HOXC10 HOXC11 HOXC12 HOXC13
HOXD1 HOXD3 HOXD4 HOXD8 HOXD9 HOXD10 HOXD11 HOXD12 HOXD13
PBX1 PBX2 PBX3 PBX4 MEIS1 MEIS2 MEIS3
HOX + TALE Gene Expression

d

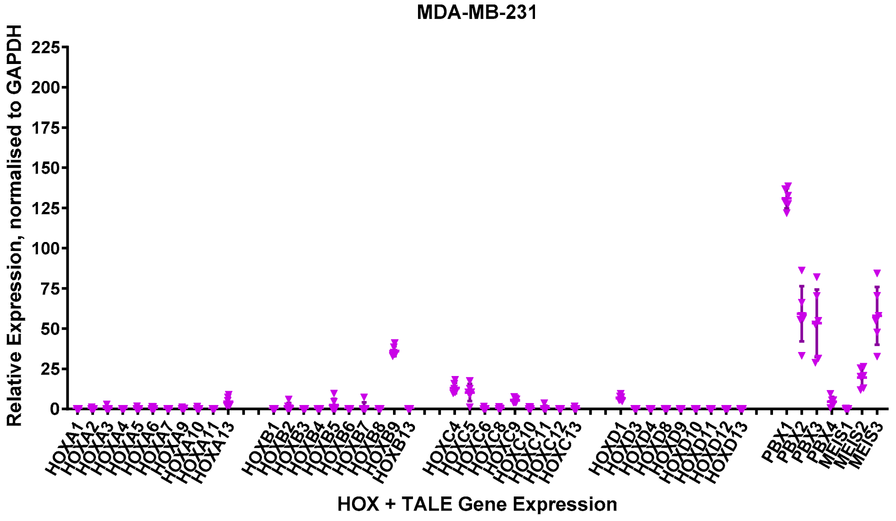
MDA-MB-231
Relative Expression, normalised to GAPDH
225
200
175
150
125
100
75
50
25
0
HOXA1 HOXA2 HOXA3 HOXA4 HOXA5 HOXA6 HOXA7 HOXA9 HOXA10 HOXA11 HOXA13
HOXB1 HOXB2 HOXB3 HOXB4 HOXB5 HOXB6 HOXB7 HOXB8 HOXB9 HOXB13
HOXC4 HOXC5 HOXC6 HOXC8 HOXC9 HOXC10 HOXC11 HOXC12 HOXC13
HOXD1 HOXD3 HOXD4 HOXD8 HOXD9 HOXD10 HOXD11 HOXD12 HOXD13
PBX1 PBX2 PBX3 PBX4 MEIS1 MEIS2 MEIS3
HOX + TALE Gene Expression

e

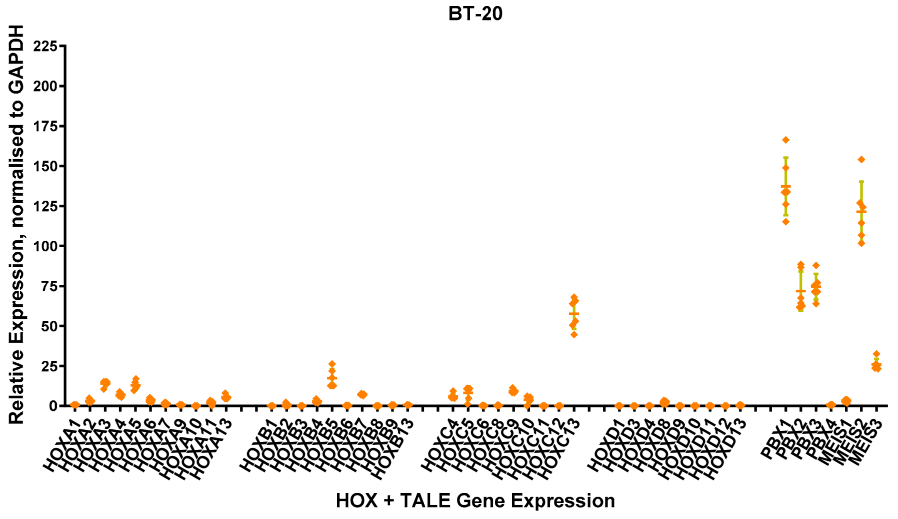
BT-20
Relative Expression, normalised to GAPDH
225
200
175
150
125
100
75
50
25
0
HOXA1 HOXA2 HOXA3 HOXA4 HOXA5 HOXA6 HOXA7 HOXA9 HOXA10 HOXA11 HOXA13
HOXB1 HOXB2 HOXB3 HOXB4 HOXB5 HOXB6 HOXB7 HOXB8 HOXB9 HOXB13
HOXC4 HOXC5 HOXC6 HOXC8 HOXC9 HOXC10 HOXC11 HOXC12 HOXC13
HOXD1 HOXD3 HOXD4 HOXD8 HOXD9 HOXD10 HOXD11 HOXD12 HOXD13
PBX1 PBX2 PBX3 PBX4 MEIS1 MEIS2 MEIS3
HOX + TALE Gene Expression

f

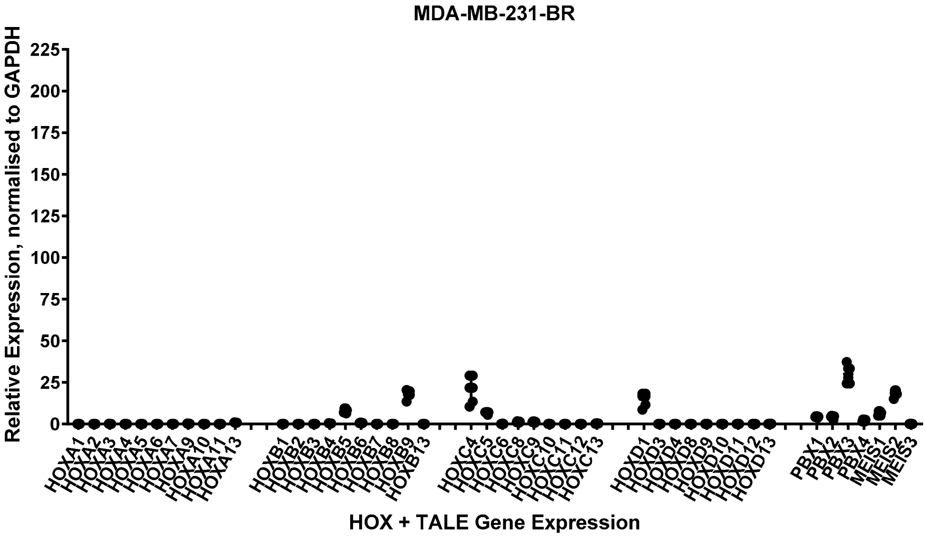

g

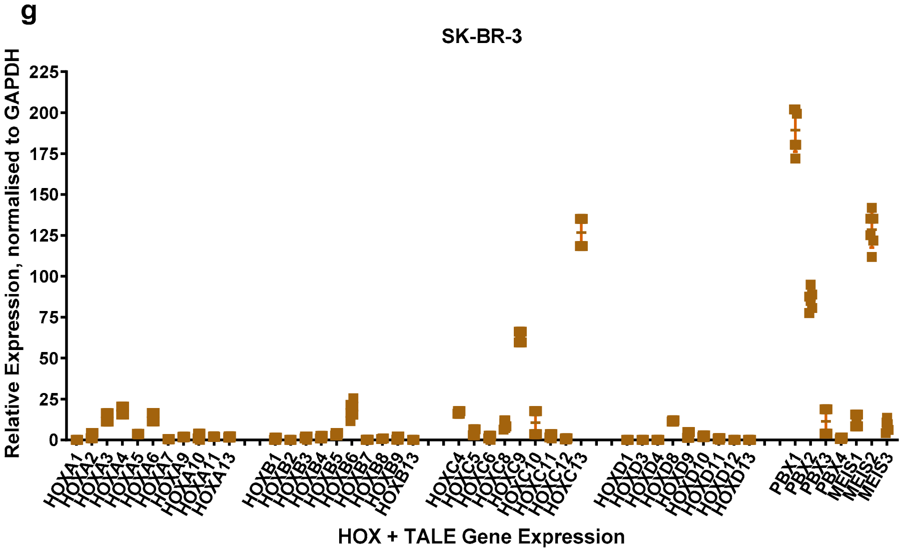

Supplemental Figure S1. HOX and TALE gene expression profiles of all 39 HOX genes and 7 TALE genes in a non-tumoregneic breast cell line (MCF10a (**a**)) and six breast cancer cell lines belonging to different molecular subclassifications; (**b**) MCF-7, derived from luminal breast cancer, (**c**) ZR-75-1, derived from luminal breast cancer, (**d**) MDA-MB-231, derived from triple negative breast cancer, (**e**) BT-20, derived from triple negative breast cancer, (**f**) MDA-MB-231-BR, derived from triple negative breast cancer, and (**g**) SK-BR-3, derived from a HER2-enriched breast cancer. Gene expression has been normalised to the housekeeping gene, GAPDH, and multiplied by a factor of 10,000. Each technical run was performed in duplicate and each biological experiment was performed in triplicate. Error bars represent the standard deviation of the mean.

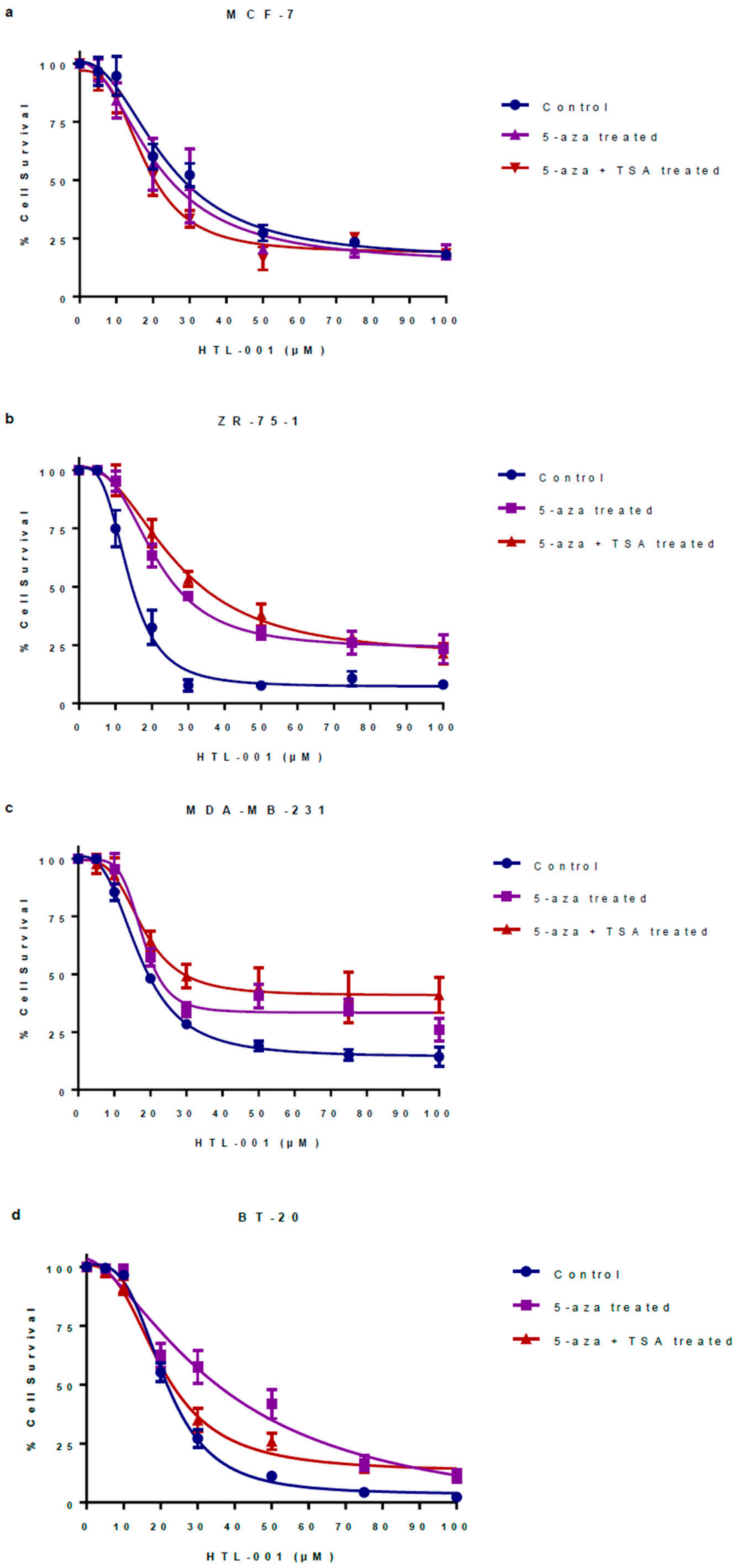

a
MCF-7
% Cell Survival
100
75
50
25
0
0 10 20 30 40 50 60 70 80 90 100
HTL-001 (µM)
Control
5-aza treated
5-aza + TSA treated
b
ZR-75-1
% Cell Survival
100
75
50
25
0
0 10 20 30 40 50 60 70 80 90 100
HTL-001 (µM)
Control
5-aza treated
5-aza + TSA treated
c
MDA-MB-231
% Cell Survival
100
75
50
25
0
0 10 20 30 40 50 60 70 80 90 100
HTL-001 (µM)
Control
5-aza treated
5-aza + TSA treated
d
BT-20
% Cell Survival
100
75
50
25
0
0 10 20 30 40 50 60 70 80 90 100
HTL-001 (µM)
Control
5-aza treated
5-aza + TSA treated

Supplemental Figure S2. HTL-001 dose response curves between control, 5-aza-treated and 5-aza + TSA-treated (a) MCF-7 cells, (b) ZR-75-1 cells, (c) MDA-MB-231 cells, and (d) BT-20 cells. Cell survival has been normalised to 100% cell survival in untreated cells (media only). Each technical run was performed in quadruplet and each biological experiment was performed in triplicate. Error bars represent the standard deviation of the mean. Statistical significance was determined by two-way ANOVA. ns (p>0.1234), * (p<0.0332), ** (p<0.0021), ***(p<0.0002), ****(p<0.0001).

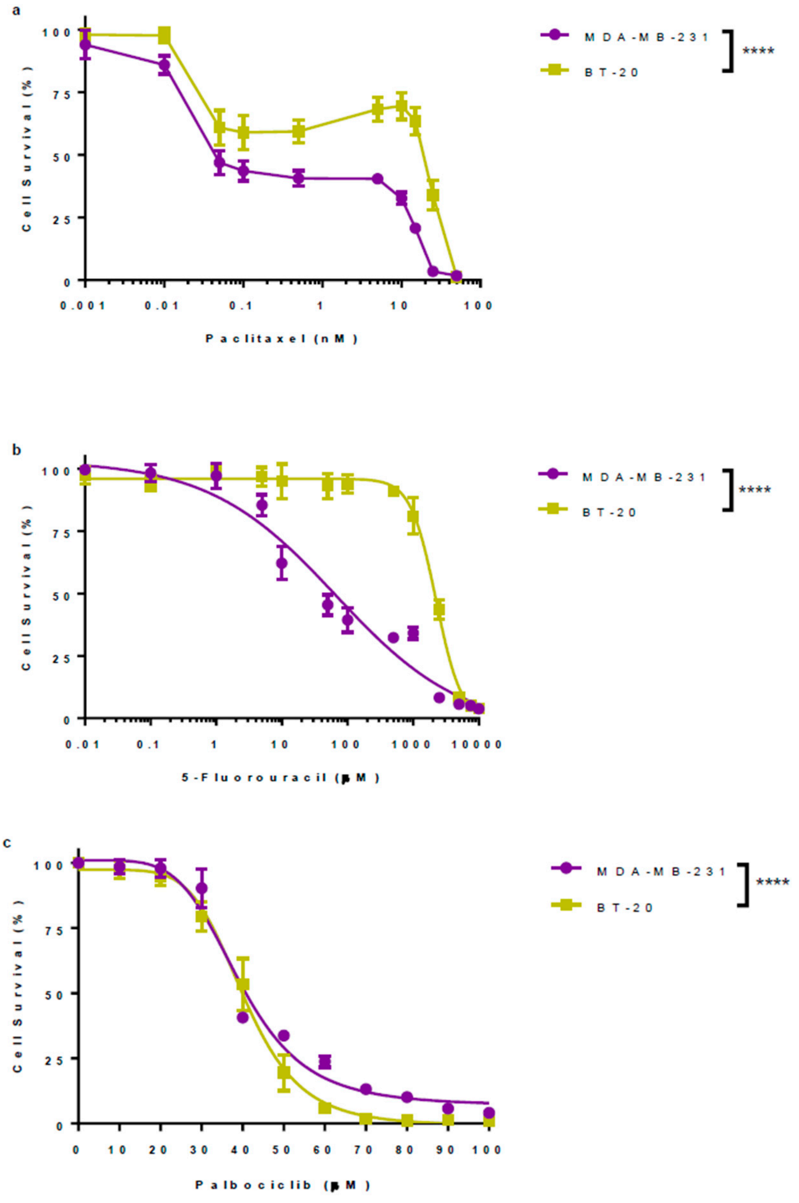

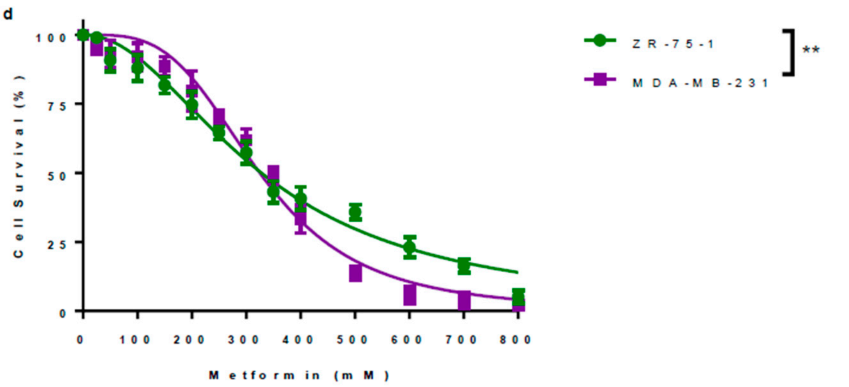

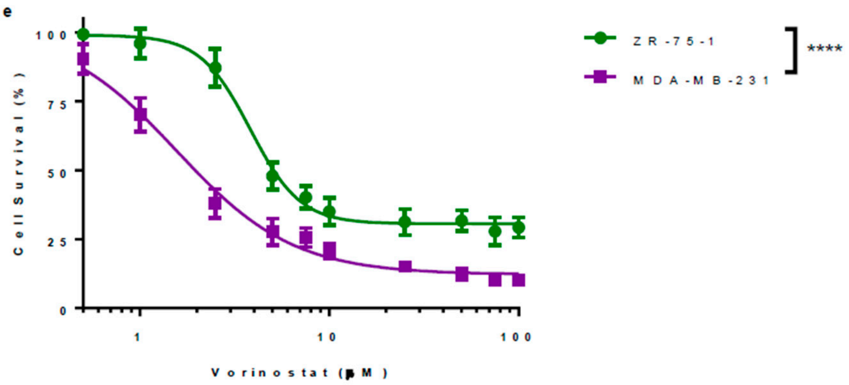

Supplemental Figure S3. Dose response curves of chemotherapeutic agents in breast cancer derived-cell lines. (a) Paclitaxel dose response curve for MDA-MB-231 (IC50 = 0.16nM) and BT-20 (IC50 = 28nM). (b) 5- Fluorouracil dose response curve for MDA-MB-231 (IC50 = 66µM) and BT-20 (IC50 = 2.2mM). (c) Palbociclib dose response curve for MDA-MB-231 (IC50 = 39µM) and BT-20 (IC50 = 40µM). (d) Metformin dose response curve for ZR-75-1 (IC50 = 328mM) and MDA-MB-231 (IC50 = 325mM). (e) Vorinostat dose response curve for ZR-75-1 (IC50 = 3.8µM) and MDA-MB-231 (IC50 = 1.6µM). Cell survival has been normalised to 100% cell survival in untreated cells (media only). Each technical run was performed in quadruplet and each biological experiment was performed in triplicate. Error bars represent the standard deviation of the mean. Statistical significance was determined by two-way ANOVA. ns ($p>0.1234$), * ($p<0.0332$), ** ($p<0.0021$), ***($p<0.0002$), ****($p<0.0001$).
